# Molecular insights into drug repurposing and drug target proteins identification for *Yersinia enterocolitica*

**DOI:** 10.1515/med-2026-1547

**Published:** 2026-10-26

**Authors:** Munazza Kanwal, Muhammad Bilal, Muhammad Faheem, Uzma Azeem Awan, Rania Ali El Hadi Mohamed, Maher S. Alwethaynani, Ammena Y. Binsaleh, Deema Fallatah, Abeer M Alghamdi, Tariq Aziz, Syed Babar Jamal

**Affiliations:** Department of Biological Sciences, National University of Medical Sciences, Rawalpindi, Pakistan; Department of Biological Sciences, Oakland University, Michigan, USA; Department of Gastroenterology, Mayo Clinic, Jacksonville, FL, USA; Department of Biology, College of Science, Princess Nourah bint Abdulrahman University, P.O.Box 84428, Riyadh 11671, Riyadh, Saudi Arabia; Department of Clinical Laboratory Sciences, College of Applied Medical Sciences, Shaqra University, Riyadh, Saudi Arabia; Department of Pharmacy Practice, College of Pharmacy, Princess Nourah bint Abdulrahman University, P.O.Box 84428, Riyadh 11671, Riyadh, Saudi Arabia; Department of Medical Laboratory, College of Applied Medical Sciences, Prince Sattam bin Abdulaziz University, Al-Kharj, Saudi Arabia; Department of Biology, Faculty of Science, Al-Baha University, Al-Baha, Saudi Arabia; Molecular Microbiology and Infectious Diseases Research Center, University of Tabuk, 71491, Tabuk, Saudi Arabia

**Keywords:** *Yersinia enterocolitica*, subtractive genomics, pan-genome analysis, drug targets, fluoroquinolone compounds, protein-protein interactions

## Abstract

**Objective:**

Infectious diseases caused by bacteria such as *Yersinia enterocolitica* pose significant global health challenges. *Y. enterocolitica*, a Gram-negative bacterium, is known for causing gastrointestinal diseases in humans. The objective of this study was to identify potential drug targets to combat *Y. enterocolitica* infections.

**Methods:**

A pan-genome analysis of 22 complete genomes of Yersinia enterocolitica was combined with subtractive genomics to identify potential drug targets. VacSol was used to screen for non-host homologous proteins, and essential proteins were identified using the Database of Essential Genes (DEG). Cytoplasmic proteins were selected based on predicted subcellular localization, modeled using SWISS-MODEL, and refined using GalaxyRefine. Their potential interactions with 30 fluoroquinolone compounds were evaluated through molecular docking using AutoDock Vina and MD simulation through GROMACS was performed.

**Results:**

The analysis identified 3,025 core genes, 2,267 non-host homologous proteins, and 29 essential proteins. Nine cytoplasmic proteins were selected for structural modeling and docking. Promising predicted interactions included the two-component response regulator OmpR with ciprofloxacin, large ribosomal subunit protein uL13 with nalidixic acid, phosphoheptose isomerase with norfloxacin, and protein-export protein SecB with ciprofloxacin.

**Conclusion:**

These findings indicated that these proteins are viable targets for drug development against *Y. enterocolitica*. The increasing antibiotic resistance of *Y. enterocolitica* highlights the urgent need for novel therapeutic strategies, making these insights crucial for future drug development.

## Introduction

Infectious diseases caused by bacteria significantly threaten global health, emphasizing the need to develop novel therapeutic and preventive strategies. *Yersinia enterocolitica* is a Gram-negative bacterium that substantially threatens public health due to its ability to cause a wide range of gastrointestinal diseases in humans [1], 2]. Furthermore, understanding the molecular mechanisms underlying the pathogenesis of *Y. enterocolitica* and identifying potential targets for drug development are paramount. *Y. enterocolitica* belongs to the genus *Yersinia*, which comprises several Gram-negative coccobacilli, three of which are well-recognised human pathogens: *Y. pestis,* the causative agent of plague, *Y. pseudotuberculosis*, and *Y. enterocolitica*. *Yersinia enterocolitica*, a member of the *Enterobacteriaceae* family, was named after Alexandre Yersin, a Swiss bacteriologist, who first identified this organism in 1894 [3], 4]. Additionally, it is a non-capsulated, non-sporing bacterium with a width of 0.5 – 0.8 μm and 1 – 3 μm in length. They have a single circular chromosome, 4,552,107 base pairs (bp) in size, and a *Yersinia* virulence plasmid (pYV), 69,704 bp long [5].

*Yersinia enterocolitica* is an asporogenous, rod-shaped bacterium that exhibits pleomorphism, ranging from small coccobacilli with rounded ends to more elongated bacilli. Additionally, the growth medium can influence the morphology of bacteria*. Y. enterocolitica* is peritrichously flagellated at 25 °C, and non-flagellated and non-motile at 37 °C [6]. There are six *Y. enterocolitica* biotypes, of which biotypes 2, 3, and 4 are the most common in humans. *Y. enterocolitic*a can be classified into approximately 60 serogroups, but only 11 of these serogroups are commonly associated with human infections. The primary serogroups responsible for yersiniosis worldwide are O:3, O:9, and O:5, with decreasing numbers of isolates belonging to serogroup O:8 [3], 7]. The pathogenesis of *Y. enterocolitica* is characterized by the ability of bacteria to translocate through the intestinal epithelium and reside in the Peyer’s patches. Additionally, Peyer’s patches provide a less hostile environment, allowing unimpeded bacterial proliferation [5]. The bacterial type III secretion system mediates this process by facilitating the translocation of toxic virulence effectors into the host cells [8]. The predominant bioserotypes associated with human yersiniosis are O:3, O:8, and O:9 [9], 10].

*Yersinia enterocolitica* was first reported in the United States in 1939 and gained attention after an outbreak in 1974 when contaminated chocolate milk led to unnecessary appendectomies in schoolchildren [11]. *Y. enterocolitica* is generally vulnerable to most antibiotics, except for beta-lactam agents such as penicillin, ampicillin, and first-generation cephalosporins [12]. However, recent studies have shown that *Y. enterocolitica* is becoming increasingly resistant to antibiotics, including amoxicillin, cefotaxime, cefuroxime, gentamicin, imipenem, meropenem, tetracycline, and tobramycin. Furthermore, chromosomally encoded β-lactamases by *Y. enterocolitica* impart resistance to all β-lactams [13]. Additionally, the prevalence of multidrug-resistant *Y. enterocolitica* strains has been reported [12], and *Y. enterocolitica* has developed resistance to fluoroquinolones. This resistance is caused by the acquisition of resistance genes through horizontal gene transfer as well as the selection pressure exerted by the misuse and overuse of antibiotics. Reports of antibiotic-resistant strains highlight the need for alternative strategies to combat *Y. enterocolitica* infections. In addition to antibiotic resistance, *Y. enterocolitica* can endure various environmental conditions, which makes it difficult to control [14]. The field of drug discovery has seen a paradigm shift towards methods based on computational biology, such as subtractive genomics. These techniques offer a powerful approach for identifying potential drug targets by analyzing the bacterial genome and proteome. This study aimed to bridge this gap by employing a unified approach of subtractive genomics to perform drug repurposing against *Y. enterocolitica*. We performed *in silico* analysis utilizing the bacterial genome and proteome to identify essential virulence factors and immunogenic proteins that can be exploited for therapeutic development. Our focus was to repurpose the drugs for which *Y. enterocolitica* was reported to be resistant. The findings of this study are sufficient for designing effective drugs to combat *Y. enterocolitica* infections.

## Materials and methods

The complete methodology is illustrated in Figure 1, and each step is elucidated in detail within the subsequent subsections.

**Figure 1:**
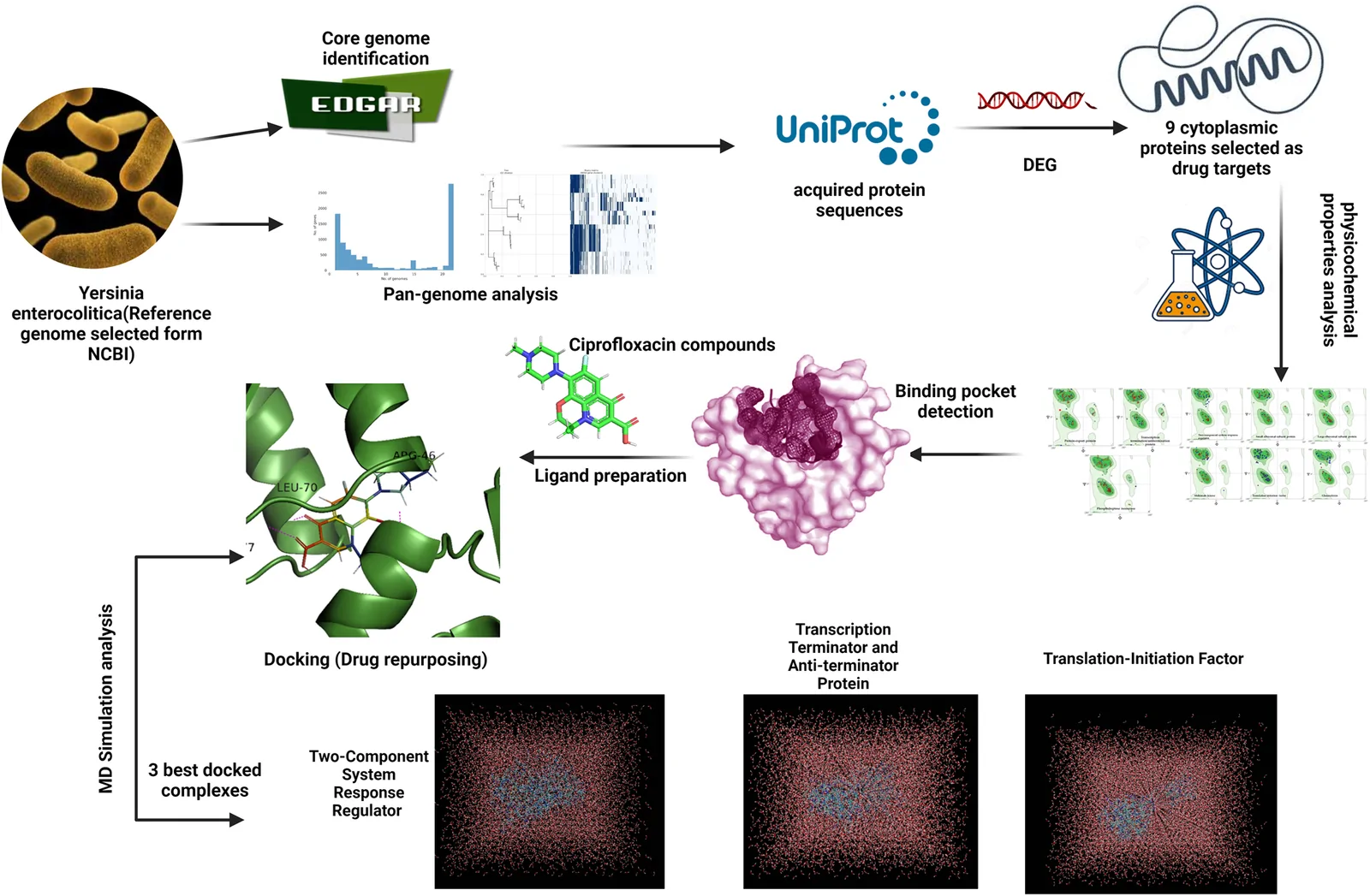
A graphical representation of the computational approach for drug repurposing in *Yersinia enterocolitica*. Complete genomes were obtained from NCBI and examined to determine the core genome. Subtractive genomics was used to filter core proteins and identify non-host homologous and critical cytoplasmic targets. Prioritized proteins were computationally modeled, screened for druggable binding sites, and docked with fluoroquinolone drugs. The top protein-ligand complexes were validated further using molecular dynamics simulations to determine interaction stability.

### Retrieval of genomic and proteomic data

In this study, we performed pan-genome analysis of 22 complete genomes downloaded from the National Center for Biotechnology Information (NCBI) (https://www.ncbi.nlm.nih.gov/). The genome FASTA sequences of the selected microorganisms were then annotated using the Prokka database, Prokka (version 1.14.6) [15], and genome-annotated files (.gff) were generated for each genome. Roary (version 3.11.2) was used for pangenome [16]. This study identified core and accessory genes that were further confirmed using EDGAR [17], and the core genome was acquired. This study did not include human subjects or patient data. All studies were carried out only on publically accessible *Y. enterocolitica* genomes. Only assemblies defined as “complete genomes” by NCBI were included to assure accuracy in comparative and pangenome analysis. Genomes that were marked as incomplete, low-quality, or without annotation were eliminated. A total of 22 genomes matched these requirements and were used for further processing.

### Selection of the core genome

The core genome is defined as the genome shared by all the strains of a specific species. It was identified using EDGAR, and the core genome file was downloaded. Reference genome **Yersinia_enterocolitica_strain_FORC_002_NZ_CP011118** was selected from EDGAR (https://edgar3. compared bin/edgar_login.cgi?cookie_test=1), as a comparison with other strains of *Y. enterocolitica.* Additionally, protein sequences were obtained from UniProt (https://www.uniprot.org/).

### Selection of non-host homologous and essential proteomes

VacSol was used to select non-host homologous proteins [18]. VacSol is a database created by the National University of Science and Technology in Pakistan. Subtractive genomic analysis was performed. The file was subsequently analyzed to identify essential proteins, which were then subjected to BLASTp analysis against the Database of Essential Genes (DEG) (http://origin.tubic.org/deg/public/index.php/blast/bacteria). Only proteins that exhibited significant hits with an E-value of 0.000001 or lower were deemed suitable for subsequent analysis [19].

### Potential virulent target identification

After identifying the essential proteins, the subsequent task involved categorizing them according to their localization and determining how they manifest themselves in a host organism. CELLO™ serves as a predictive tool and aids in the efficient design of drugs and metabolic pathways [20].

### Homology modeling, catalytic pocket detection, structural analysis

Following protein prioritization, their physicochemical activities were checked using ProtParam (https://web.expasy.org/protparam) using default parameters. The instability index was calculated, and GRAVY was analyzed. Modeling was performed using the Swiss model (https://swissmodel.expasy.org), which is based on homology modeling, and 3D structures were acquired. Additionally, the DoGSite Scorer (https://proteins.plus/help/dogsite)was used for catalytic pocket detection and to evaluate the druggability of the 3D structure. The catalytic sites are those to which the ligands can bind during docking. Only proteins that were suitable as drug targets or present in the cytoplasmic region were used. Furthermore, the protein structure was validated using the Galaxy Refine (https://galaxy.seoklab.org/cgi-bin/submit.cgi?type=REFINE), Ramachandran plot (https://swissmodel.expasy.org/), and the ERRAT (https://www.doe-mbi.ucla.edu/errat/) score was calculated [21].

### Ligand’s preparation

A review of the literature resulted in the compilation of a list of 30 fluoroquinolone ligands. The corresponding 2D structure for each ligand was acquired in SDF format from the PubChem database (https://pubchem.ncbi.nlm.nih.gov). These SDF files were imported into Discovery Studio Visualiser (BIOVIA), where they were utilised to generate 3D structures and check that their geometry was accurate. Hydrogen atoms were incorporated if necessary, and bond lengths and angles were refined by structural optimisation. The ligands were subsequently saved in PDB format for further docking studies [22].

### Molecular docking analysis

Molecular docking is an effective approach for identifying the structural and energetic bases of the interaction between macromolecules and substrates, endogenous and exogenous ligands, and inhibitors. This technique plays an essential role in accelerating the screening of large libraries of compounds for drug development [23]. Therefore, AutoDock Vina significantly improves the molecular docking speed and accuracy by using a new scoring function, efficient optimization, and multithreading on multicore machines [24]. AutoDock was used for pdbqt file preparation for both the ligands and receptors. The PDBQT files for both receptors and ligands were generated using AutoDock Tools (ADT, version 1.5.7). The target protein structure in PDB format was initially imported into ADT for receptor preparation. Gasteiger charges were assigned, polar hydrogen atoms were introduced, and water molecules and any heteroatoms were eliminated. The structure was subsequently saved in PDBQT format. To prepare ligands, each ligand structure (originally in PDB format) was imported into ADT. Torsion tree roots were established, rotatable bonds were identified, Gasteiger charges were assigned, and non-polar hydrogens were merged. The ligands were subsequently saved in PDBQT format, ensuring that they were prepared for docking. Moreover, active sites were determined, a grid was set for the receptor to attach efficiently, all ligands were docked one by one, and the best-docked complex was determined.

### Molecular dynamic simulation

The GROMACS version 2024.02 program (General Public License; http://www.gromacs.org) and the CHARMM36 force field [25] were utilized to choose the optimal docking conformation of the ligand with the target protein for a 100 ns molecular dynamics simulation. The system was prepared for simulation through an initial equilibration in the NVT ensemble (constant particle number, volume, and temperature). A V-rescale thermostat was employed to maintain the temperature at 303.15 K to ensure thermal stability. The system may reach the target temperature before the production phase through a 1 ns equilibration process. The transferable intermolecular potential with a three-point (TIP3P) water model was employed in a cubic box measuring 100 × 100 × 100 Å, ensuring a minimum distance of 10 Å between the protein and each side of the three-dimensional box for each ligand–protein combination [26]. The CHARMM General Force Field (CGenFF) tool [27] was used to automatically compute the CHARMM force field parameters for the examined ligands under the ParamChem project (https://cgenff.umaryland.edu/). Using the Monte Carlo ion-placing technique [28], the protein residues were designated their standard ionization states at physiological pH (7.0), and the whole complexes were neutralized with the requisite amounts of K+ and Cl− ions. The molecular dynamics simulation was conducted in three phases, employing a force constant of 1,000 kJ/mol nm^2^ to constrain all heavy atoms and maintain the original protein structure [29]. The initial phase involved employing the steepest descent approach to optimize the configuration of each system over 5,000 iterations (5 ps). The system underwent two-step equilibration, which was conditioned for 100,000 iterations (100 ps) at each stage. The initial equilibration phase was conducted under a constant number of particles, volume, and temperature (NVT) ensemble using the Berendsen temperature coupling method to maintain the temperature within the three-dimensional box [30]. The second equilibration stage was conducted using a Parrinello-Rahman barostat [31] under a constant number of particles, pressure, and temperature (NPT) ensemble at 1 atm and 303.15 K. The molecular dynamics simulation was conducted with a time step of two femtoseconds (fs) for a duration of two hundred thousand nanoseconds during the production phase. The application of periodic boundary conditions (PBC) mitigated the edge effects and simulated an infinite system, thereby enhancing the veracity of molecular interactions. Electrostatic interactions were calculated using the Particle Mesh Ewald (PME) method to ensure the precise handling of long-range electrostatic forces. Non-bonded interactions were restricted to 1.2 nm (nm), thus optimizing the computational performance and ensuring precision in short-range interactions. Molecular dynamics simulations were conducted for 100 ns under constant pressure (NPT ensemble) using the PME approach for long-range electrostatic interactions [32]. Employing a rapid and precise technique to address long-range Coulomb interactions might facilitate the attainment of stable nanosecond trajectories in highly polar biomolecules, such as proteins. The employed LINCS approach constrained all covalent bond lengths, including hydrogen bonds, thereby facilitating a 2fs integration time step [33]. The Verlet cutoff technique was employed to truncate non-bounded interactions – Coulomb (electrostatic potential) and Lennard-Jones (Pauli repulsion and hydrophobic/van der Waals attractions) – at 10 Å [34]. The protein and ions were propelled using the CHARMM36m all-atom force field during molecular dynamic simulation [35]. The examination of MD trajectories using GROMACS’ integrated features facilitated comparative data evaluation, including RMSD and radius of gyration (Rg). The Distance Calculation Tool from the Visual Molecular Dynamics 1.9.3 (VMD) package, created by the University of Illinois at Urbana-Champaign, was employed to evaluate the fluctuations in distance between specified ligand/protein atoms during the simulation period [36]. This method enabled the monitoring and investigation of potential interactions between the ligand and critical protein residues. The binding free energy between the ligand and protein was calculated using the GROMACS MD engine (mdrun). Data of ligand–protein conformational analyses were generated using PyMOL, a graphical software (version 2.0.6) (SchrödingerTM, NY, USA) [37].

## Results

### Retrieval of genomic data and pan-genome analysis

Genomic data was obtained from the NCBI, a renowned repository for biological information. A total of 23 complete genomes were identified during the retrieval process; however, only 22 were found to have a complete and accurate genome in the database. The 23rd **genome assembly, ASM2534022v1,** lacks a complete genome status. Genomic annotation was performed using PROKKA, which generated multiple files. Specifically, the GFF files were generated. Subsequently, pan-genomic analysis was conducted using Roary on a Linux system, which yielded several results. These outcomes included gene frequency results, a pie chart illustrating genome frequency, and a matrix file representing the pan-genome. The results are presented in Figure 2A–C.

**Figure 2:**
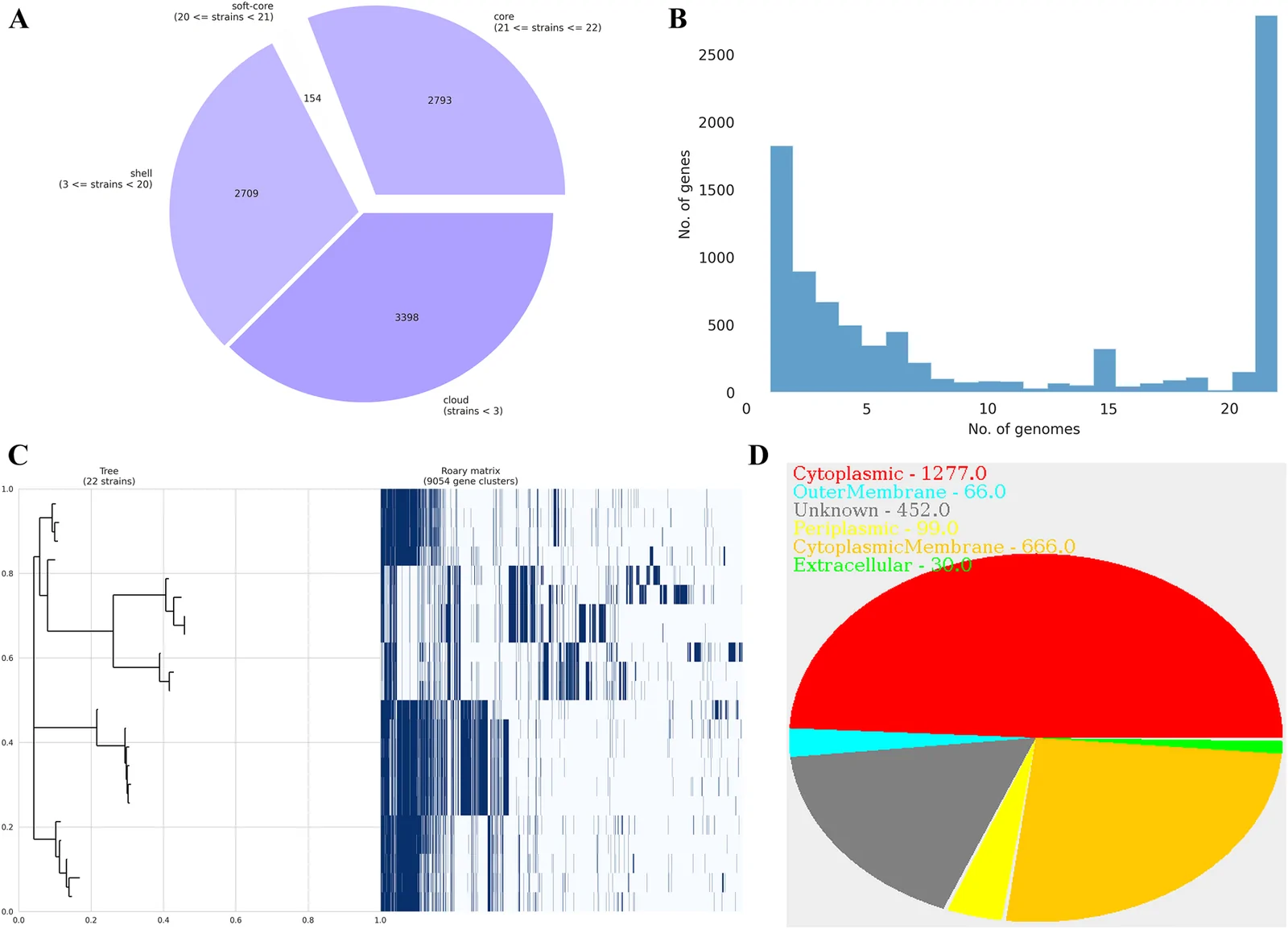
Four panels show gene counts across 22 genomes, gene-family prevalence, a clustered heatmap, and protein subcellular localization. A. The bar graph represents the relationship between the number of genes present in each of the 22 genomes considered. B. The pie chart illustrates the distribution of gene families across 22 strains, categorized into four groups based on their prevalence. The **core genome** (2,793 genes) includes genes present in all or nearly all strains (21–22 strains). The **soft-core genome** (154 genes) comprises genes found in 20 strains. The **shell genome** (2,709 genes) includes genes present in 3–19 strains, while the **cloud genome** (3,398 genes) represents rare genes found in fewer than 3 strains. This distribution highlights a substantial accessory genome, suggesting high genomic diversity and adaptability within the species. C. The provided image depicts a clustered heatmap integrated with dendrograms, which is a widely employed visualization technique for representing clustered data, particularly in the domain of gene expression or biological datasets. Dendrograms, both vertical and horizontal, visually represent the hierarchical clustering of rows and columns. These dendrograms provide insights into the similarities between different clusters. The cells in the heatmap indicate a magnitude of a specific variable, such as the level of gene expression. The intensity of the color used in the cells reflects this magnitude. The labels associated with rows and columns are likely identifiers used to cluster the data. The annotations indicated that the rows in the dataset were likely associated with genes or proteins. However, the columns appear to be arranged according to different time points. The use of images plays a crucial role in the investigation of gene expression patterns, the identification of gene clusters, and the comprehension of biological pathways. D. The pie chart generated by VacSol shows different localizations of proteins based on their presence, and different color representations are used to describe their locations.

Furthermore, pan-genome analysis was conducted using the EDGAR tool to provide additional validation. The resulting core genome file, which contained 4,213 genes, was downloaded. Subsequently, duplicated genes were identified and removed, resulting in a final count of 3,025 core genes.

### Drug target identification (proteomic data retrieval, identification of non-host homologous and essential genes, and protein localization)

The proteins comprising the core genome were subjected to UniProt and their fully reviewed sequences were obtained. Non-host homologous proteins were characterized using VacSol. Notably, this software operates based on the principles of BLAST, resulting in the identification of 2,267 non-host homologous proteins. Figure 2D illustrates the results obtained using VacSol. The Database of Essential Genes (DEG) (https://tubic.org/deg/public/index.php/blast/bacteria) was used to identify essential proteins. The parameters were set as follows: the percentage had to be greater than 35 %, the alignment length should be above 100, and the number of mismatches should be less than 10. It has been determined that a total of 29 proteins have been identified as essential for the survival and growth of *Y. enterocolitica*. The 29 proteins were submitted to the subcellular localization predictive system (CELLO) for subcellular localization analysis to identify proteins that belong to specific regions. This study aimed to identify potential candidates for drug development. As a result of this characterization, 9 cytoplasmic proteins and 1 membrane protein were detected.

### Potential virulent target identification and binding pocket detection

To identify virulence targets, a set of nine cytoplasmic proteins was selected for analysis. These proteins were subjected to ProtParm analysis, a tool used to assess their physicochemical properties. Specifically, the instability index of each protein was determined, and proteins with an instability index greater than 40 were classified as unstable. In addition, the grand average of hydropathy (GRAVY) was calculated for each protein. Moreover, the modeling process was executed using the Swiss Model, which allowed the construction of PDB structures.

Additional validation was conducted by assessing the ERRAT score, which measures the overall quality of the protein structure, and by constructing Ramachandran plots, which provide insights into the conformational quality of the protein backbone, as presented in Figure 3. Each protein assessed in this study exhibited a commendable ERRAT score exceeding 90. Notably, using the Ramachandran plot, we observed that the Large Ribosomal Subunit protein achieved the highest score of 99.29 %, while the transcription terminator and anti-terminator proteins attained the lowest score of 91.48 %. The remaining proteins were within this range. The binding pockets were identified using DogGSite Scorer. According to our analysis, the transcription terminator, anti-terminator protein, and translation initiation factor exhibited the most significant pocket score of 0.8.

**Figure 3:**
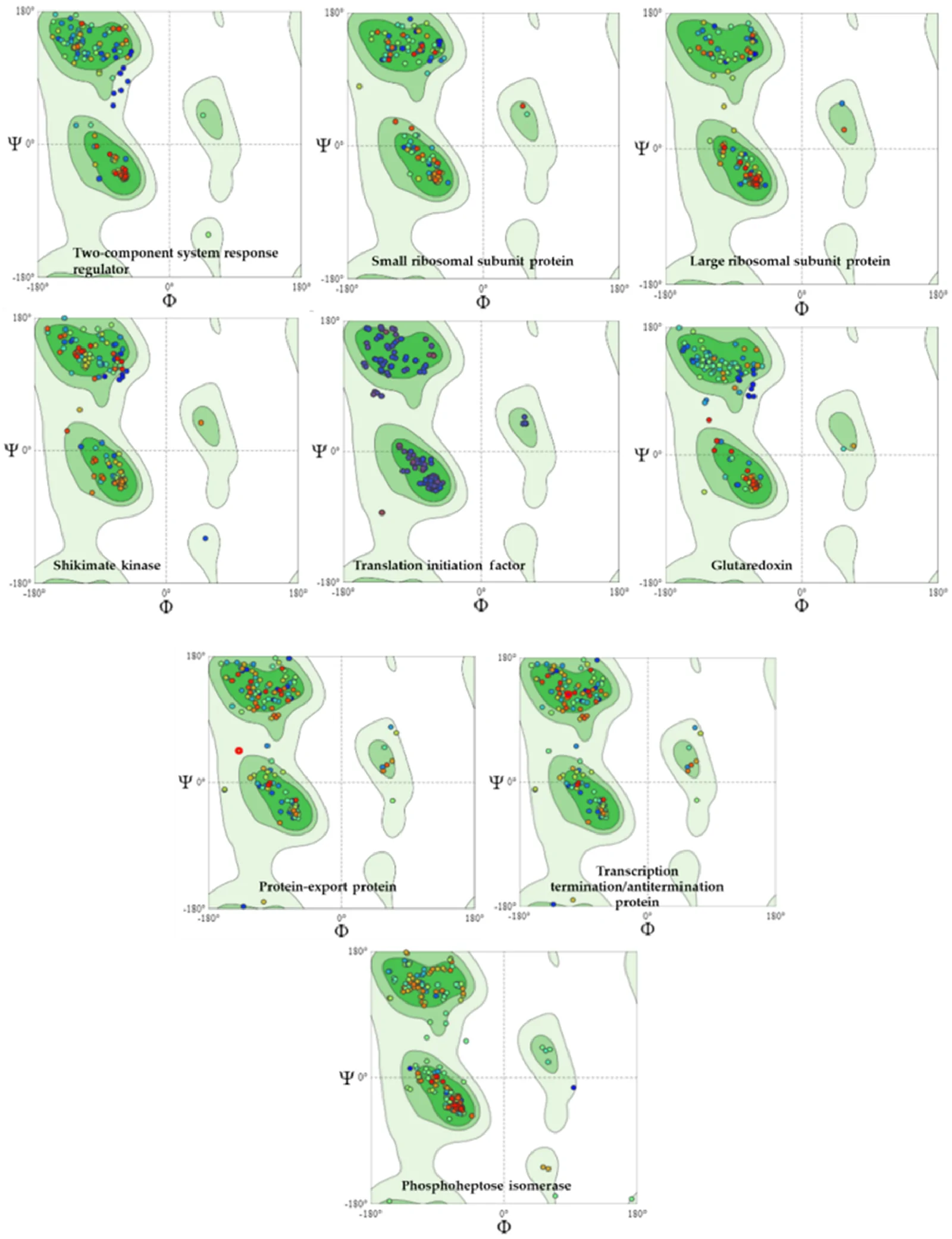
Shows Ramachandran plots for each target protein. The first quadrant, known as quadrant I, represents the region of confirmation in which all residues are permitted. A left-handed alpha band was observed in this region. The region known as quadrant II is the largest in the graph. In particular, the conditions at this location are excellent for atom confirmation. Quadrant III, the largest region following quadrant II, holds this distinction owing to its size. Within this particular geographical area, it has been observed that the prevalence of individuals exhibiting right-handedness is relatively high. This phenomenon is commonly referred to as right-handed alpha. There is hardly any framed location in quadrant IV. The conformation characterized by a torsion angle psi (ψ) ranging from −180 to 0 °and another torsion angle phi (φ) ranging from 0° to 180° is unfavorable, primarily because of steric conflict.

### Molecular docking analysis

The molecular docking technique was used to determine Root Mean Square Deviation (RMSD) and docking scores. The study was conducted using the AutoDock Vina software. Thirty antibiotic compounds belonging to the fluoroquinolone class were considered. The ligand structures were obtained from PubChem. All nine proteins exhibited favorable interactions with the ligands, as shown in Table 1. Moreover, among these proteins, the large ribosomal subunit protein demonstrated the highest level of interaction, as indicated by a binding energy of −7.8 kcal/mol, and a root mean square deviation (RMSD) value of 0.000 when docked with nalidixic acid. Several studies have investigated the antibacterial activity of nalidixic acid in *Y. enterocolitica*. According to existing research, it has been observed that *Y. enterocolitica* strains, which were previously susceptible to nalidixic acid, have shown an increase in resistance in recent years. This increase in resistance can be attributed to selective pressure created by the use of fluoroquinolones in both humans and animals [38], 39].

**Table 1:** Indicates the results of molecular docking obtained after interaction of proteins with certain drug compounds, along with their docking score, binding energy and RMSD values.

Protein	Antibiotic compound	Number of interactions	Binding energy	RMSD for best pose
Large-ribosomal subunit protein	Nalidixic acid	11	−7.8 (kcal/mol)	0.000
Two-component system response regulator	Ciprofloxacin	14	−7.4 (kcal/mol)	0.000
Protein-export protein	Ciprofloxacin	8	−7.3 (kcal/mol)	0.000
Glutaredoxin	Grepafloxacin	14	−7.2 (kcal/mol)	0.000
Small-ribosomal subunit protein	Fleroxacin	9	−6.3 (kcal/mol)	0.000
Transcription termination/antitermination protein	Ofloxacin	6	−6.3 (kcal/mol)	0.000
Phosphoheptose isomerase	Norfloxacin	9	−5.4 (kcal/mol)	0.000
Translation initiation factor	Ciprofloxacin	13	−5.4 (kcal/mol)	0.000
Shikimate kinase	Ciprofloxacin	11	−4.5 (kcal/mol)	0.000

Protein Shikimate kinase exhibited the fewest interactions with ciprofloxacin, as indicated by the binding energy of −4.5 kcal/mol, and the RMSD value was 0.6835 for our considered best-docked pose. According to the current medical guidelines, ciprofloxacin is the recommended treatment for enterocolitis caused by *Y. enterocolitica*. In cases where the infection is more severe, a combination of the two antibiotics is suggested for optimal efficacy. In severe infections, ciprofloxacin is usually combined with an aminoglycoside, such as gentamicin or a third-generation cephalosporin [40].

All of these cytoplasmic proteins were studied in detail, and docking was performed with shortlisted fluoroquinolone compounds. The results for large ribosomal subunit proteins are presented in Figure 4(a and b) and Table 2. Similarly, Figure 5(a and b) and Table 3 elaborate on phosphate isomerase and its interaction with the top five antibiotic compounds. Moreover, the docking results for the Protein-export protein are shown in Figure 6 and Table 4. Figure 7(a and b) and Table 5 present the data for the interaction of Shikimate kinase. Furthermore, Figure 8(a and b) through Figure 9(a and b) and Tables 6–10 represent data for the interaction of the Translation initiation factor, Glutaredoxin, Small ribosomal subunit protein (Figure 10), Transcription terminator and Anti-terminator protein (Figure 11), and Two-Component System Response Regulator protein (Figure 12), respectively.

**Figure 4:**
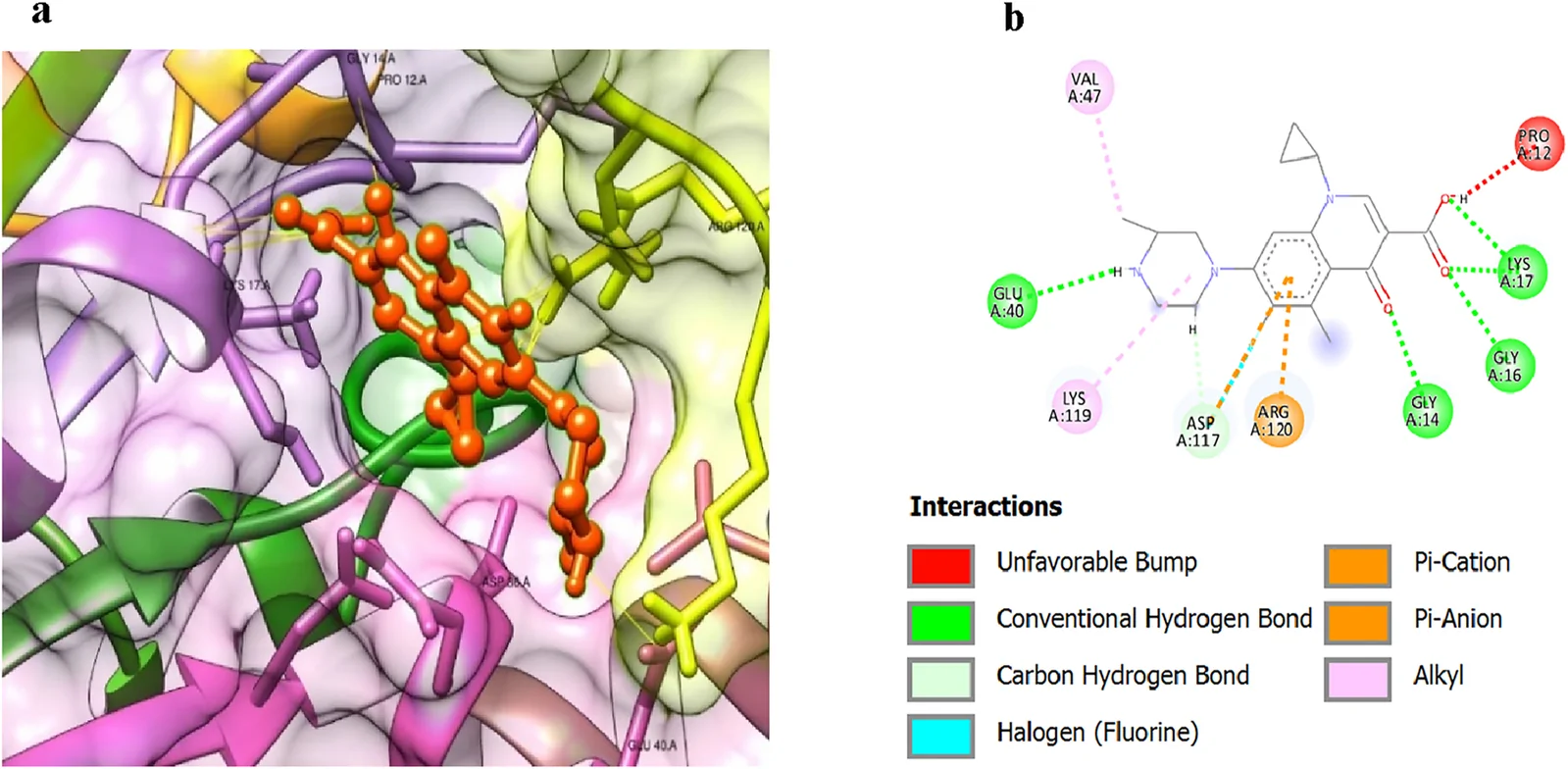
Predicted binding interactions between nalidixic acid and the large ribosomal subunit protein. (A) Three-dimensional view of nalidixic acid within the protein binding pocket. (B) Two-dimensional interaction map showing contacts between nalidixic acid and surrounding amino acid residues, with interaction types distinguished by color.

**Table 2:** Docking results of large ribosomal subunit protein with different antibiotic compounds.

Protein	Antibiotic compound	Number of interactions
Large-ribosomal subunit protein	Nalidixic acid	11
	Ofloxacin	4
	Pipemidic acid	6
	Tosufloxacin	6
	Besifloxacin	6

**Figure 5:**
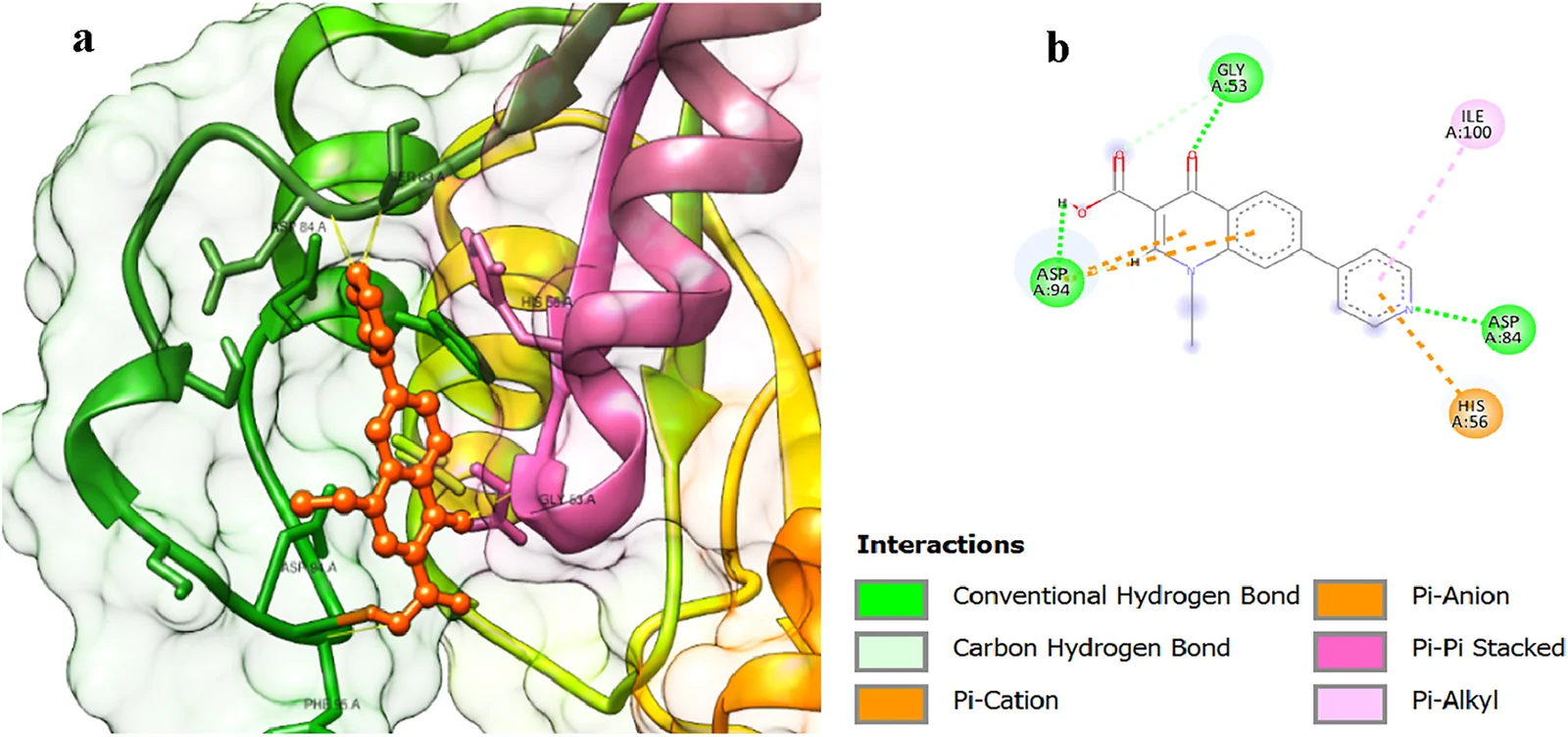
Predicted binding interactions between norfloxacin and phosphoheptose isomerase. (A) Three-dimensional view of norfloxacin within the protein binding pocket. (B) Two-dimensional interaction map showing contacts between norfloxacin and surrounding amino acid residues, with interaction types distinguished by color.

**Table 3:** Docking result of phosphoheptose isomerase with different antibiotic compounds.

Protein	Antibiotic compound	Number of interactions
Phosphoheptose isomerase	Norfloxacin	9
	Rufloxacin	4
	Tosufloxacin	7
	Sparfloxacin	3
	Nadifloxacin	5

**Figure 6:**
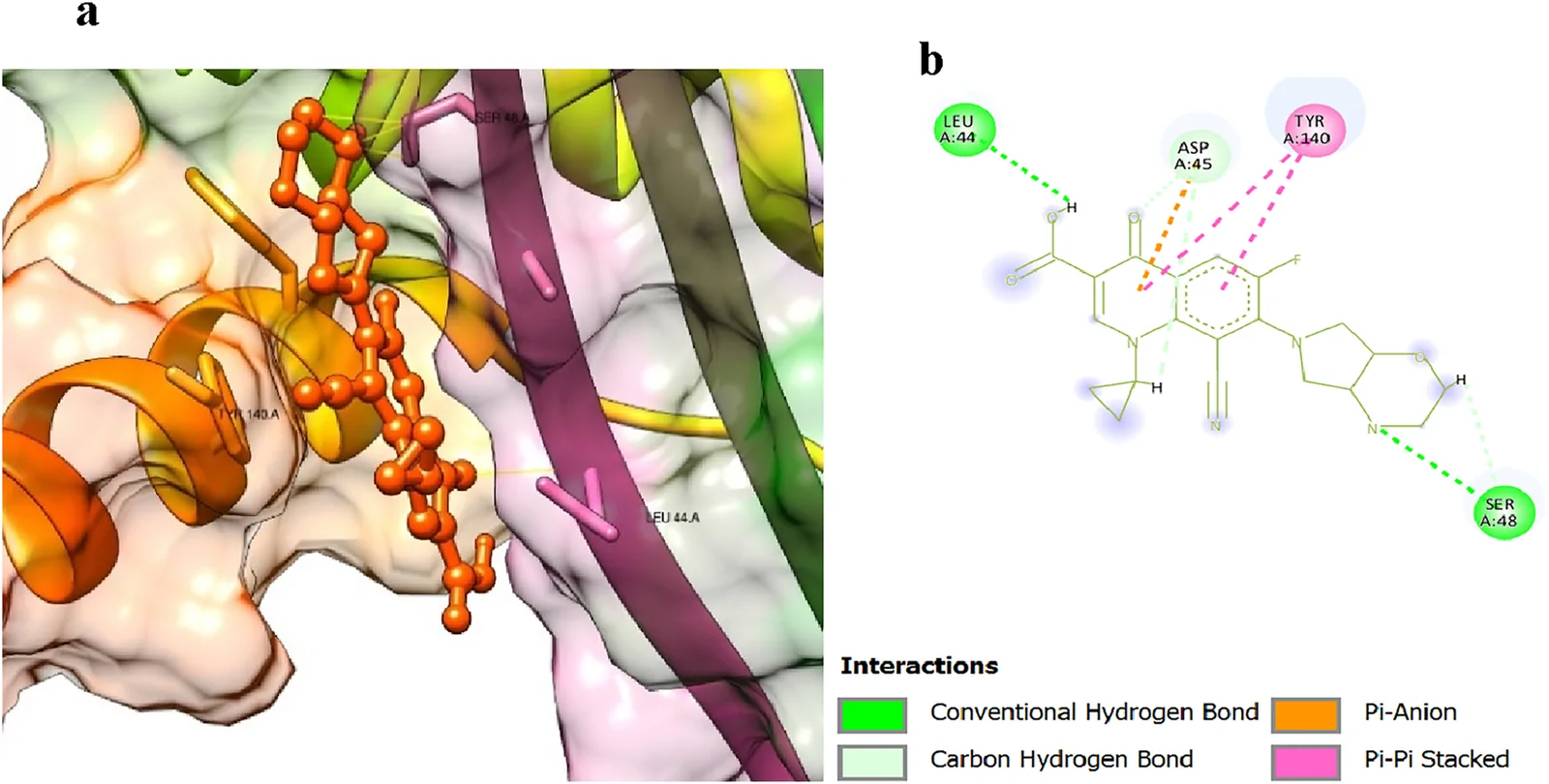
Predicted binding interactions between ciprofloxacin and the protein-export protein SecB. (A) Three-dimensional view of ciprofloxacin within the protein binding pocket. (B) Two-dimensional interaction map showing contacts between ciprofloxacin and surrounding amino acid residues, with interaction types distinguished by color.

**Table 4:** Docking result of protein-export protein with different antibiotic compounds.

Protein	Antibiotic compound	Number of interactions
Protein-export protein	Ciprofloxacin	8
	Moxifloxacin	3
	Clinafloxacin	3
	Piromidic acid	4
	Gatifloxacin	3

**Figure 7:**
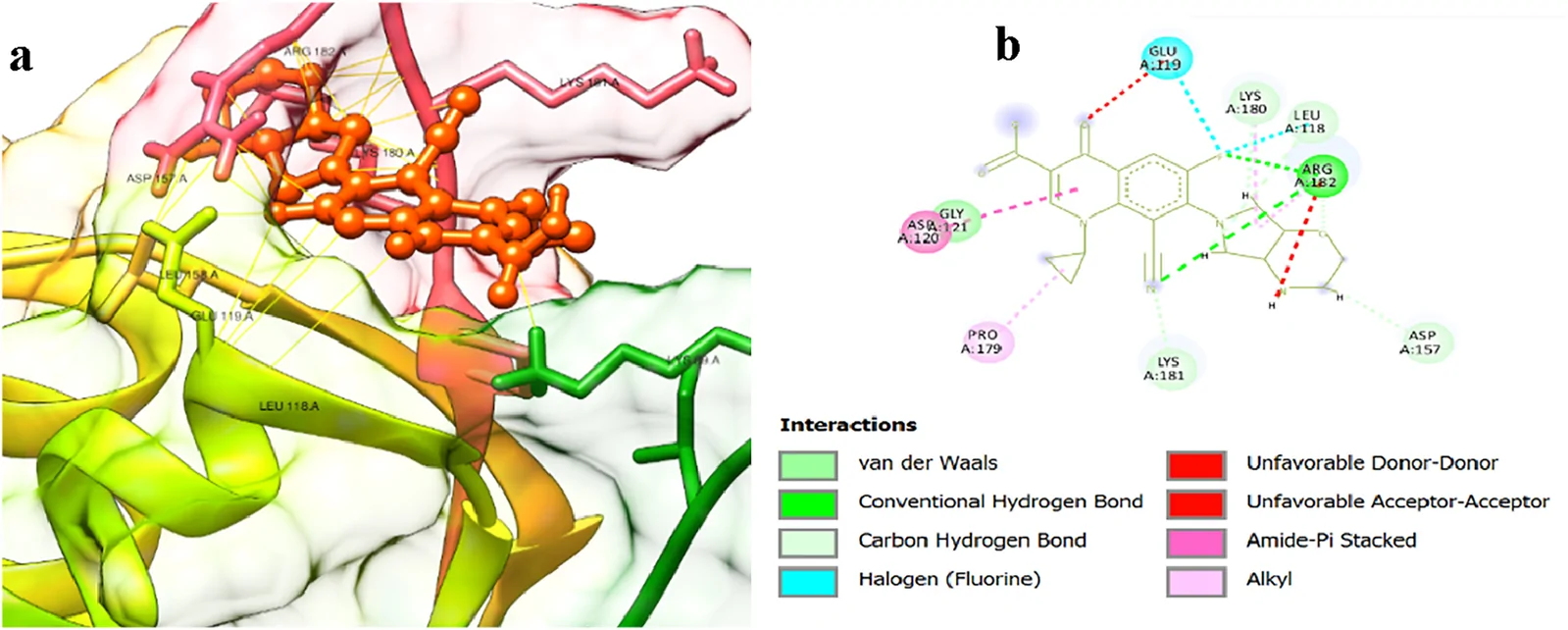
Predicted binding interactions between ciprofloxacin and shikimate kinase. (A) Three-dimensional view of ciprofloxacin within the protein binding pocket. (B) Two-dimensional interaction map showing contacts between ciprofloxacin and surrounding amino acid residues, with interaction types distinguished by color.

**Table 5:** Docking results of Shikimate kinase with different antibiotic compounds.

Protein	Antibiotic compound	Number of interactions
Shikimate kinase	Ciprofloxacin	11
	Balofloxacin	3
	Ofloxacin	4
	Lomefloxacin	2
	Pefloxacin	2

**Figure 8:**
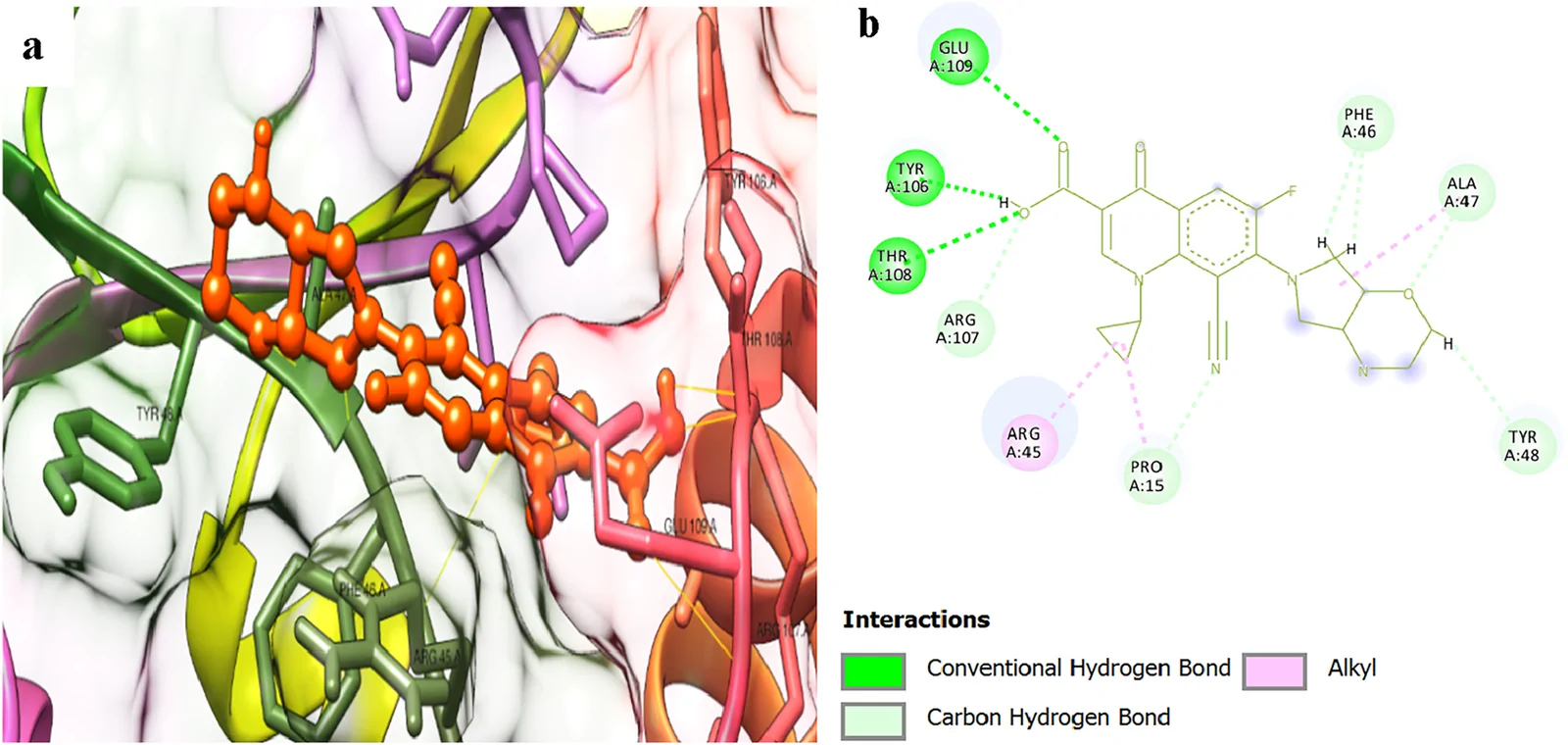
Predicted binding interactions between ciprofloxacin and translation initiation factor. (A) Three-dimensional view of ciprofloxacin within the protein binding pocket. (B) Two-dimensional interaction map showing contacts between ciprofloxacin and surrounding amino acid residues, with interaction types distinguished by color.

**Figure 9:**
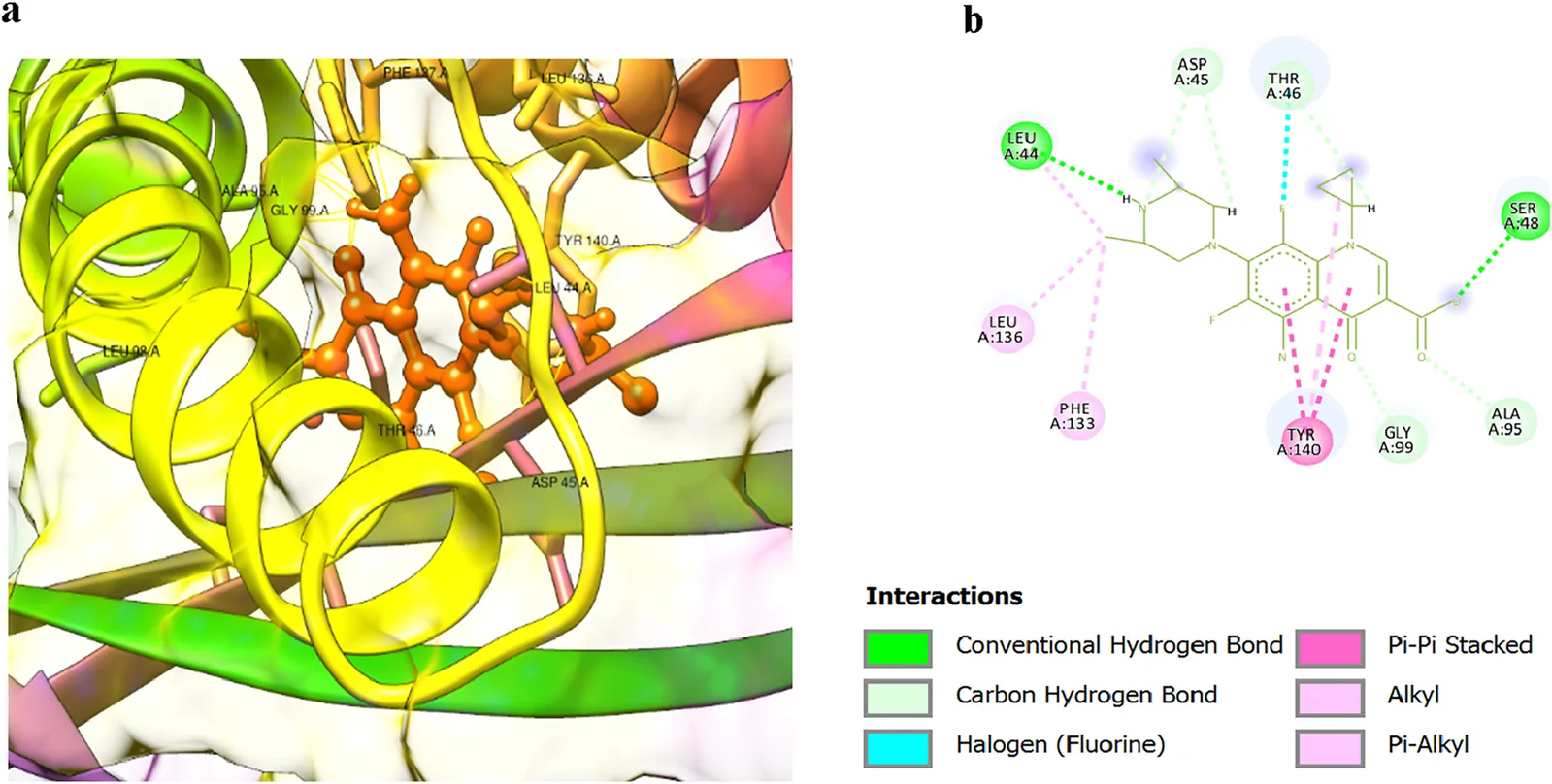
Predicted binding interactions between grepafloxacin and glutaredoxin. (A) Three-dimensional view of grepafloxacin within the protein binding pocket. (B) Two-dimensional interaction map showing contacts between grepafloxacin and surrounding amino acid residues, with interaction types distinguished by color.

**Table 6:** Docking results of translation initiation factor with different antibiotic compounds.

Protein	Antibiotic compound	Number of interactions
Translation-initiation factor	Ciprofloxacin	13
	Gatifloxacin	4
	Rufloxacin	5
	Besifloxacin	4
	Grepafloxacin	4

**Table 7:** Docking results of Glutaredoxin with different antibiotic compounds.

Protein	Antibiotic compound	Number of interactions
Glutaredoxin	Grepafloxacin	14
	Ciprofloxacin	3
	Rufloxacin	4
	Clinafloxacin	3
	Piromidic acid	3

**Table 8:** Docking results of the small ribosomal subunit protein with different antibiotic compounds.

Protein	Antibiotic compound	Number of interactions
Small ribosomal subunit protein	Fleroxacin	9
	Norfloxacin	3
	Pefloxacin	4
	Rufloxacin	3
	Gemifloxacin	4

**Table 9:** Docking results of the transcription terminator and Anti-terminator protein with different antibiotic compounds.

Protein	Antibiotic compound	Number of interactions
Transcription termination/antitermination protein	Ofloxacin	6
	Ciprofloxacin	3
	Enoxacin	4
	Balofloxacin	4
	Besifloxacin	4

**Table 10:** Docking results of the two-component system response regulator with different antibiotic compounds.

Protein	Antibiotic compound	Number of interactions
Two-component system response regulator	Ciprofloxacin	14
	Enrofloxacin	3
	Enoxacin	5
	Pefloxacin	4
	Nadifloxacin	3

**Figure 10:**
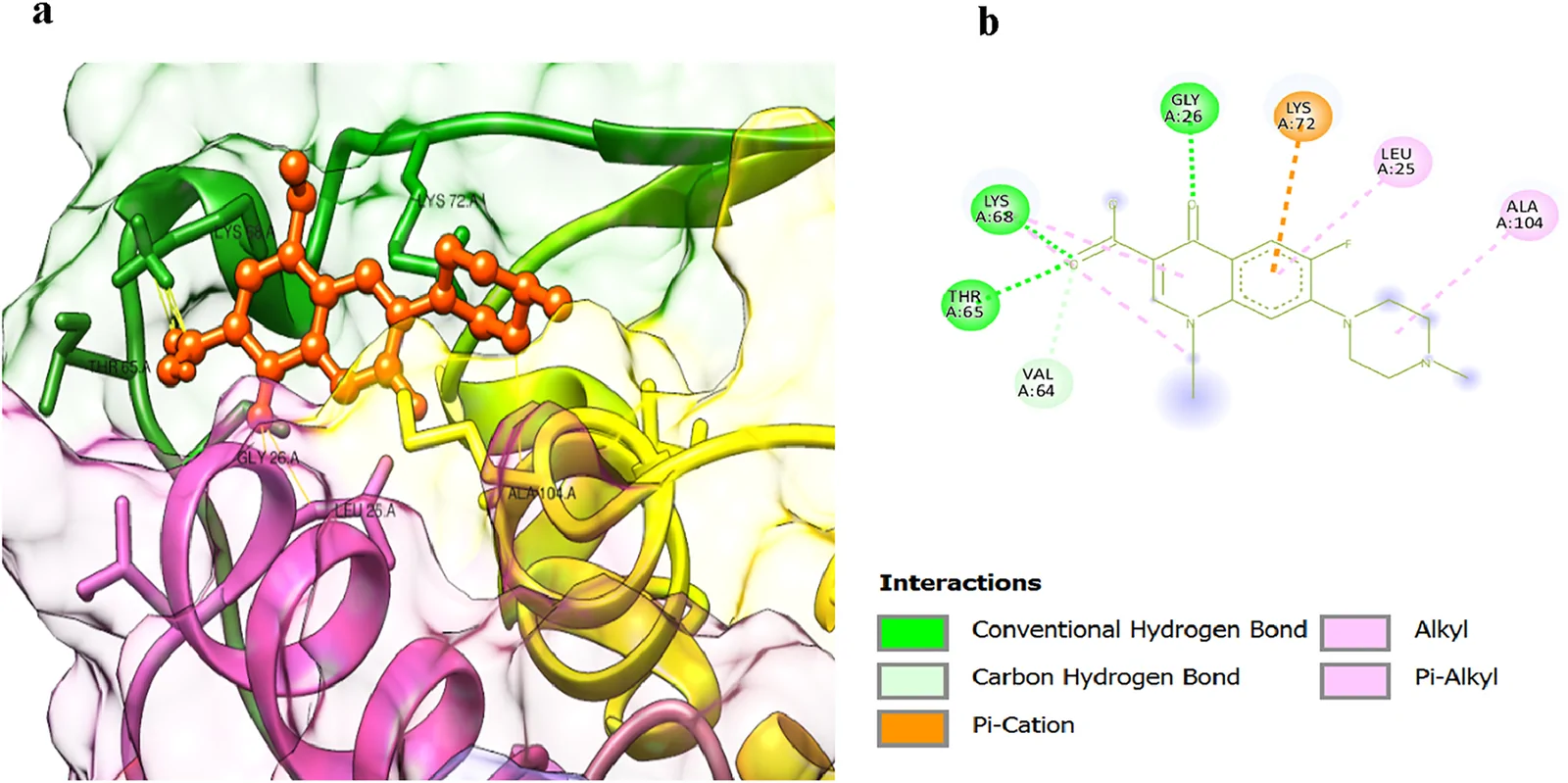
Predicted binding interactions between fleroxacin and the small ribosomal subunit protein. (A) Three-dimensional view of fleroxacin within the protein binding pocket. (B) Two-dimensional interaction map showing contacts between fleroxacin and surrounding amino acid residues, with interaction types distinguished by color.

**Figure 11:**
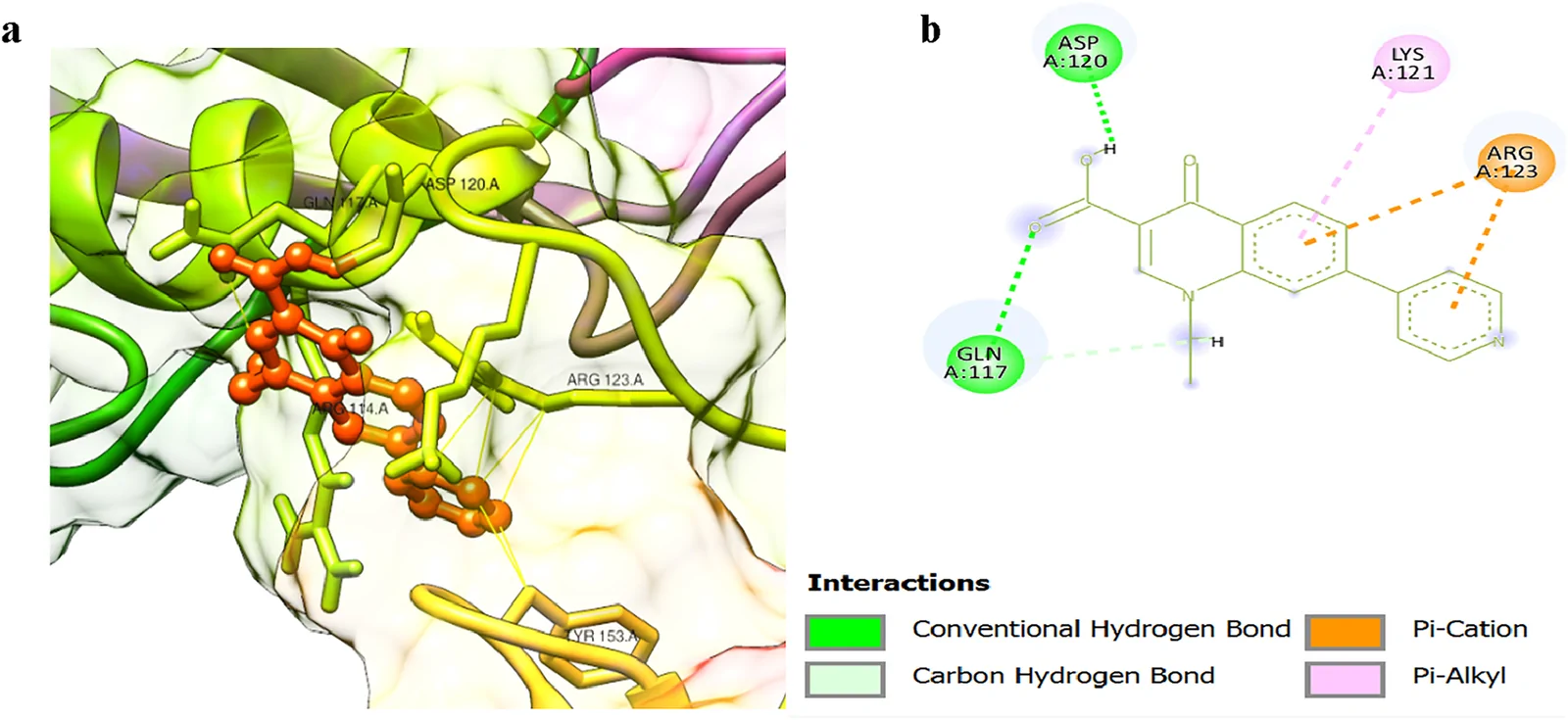
Predicted binding interactions between ofloxacin and the transcription terminator/antiterminator protein. (A) Three-dimensional view of ofloxacin within the protein binding pocket. (B) Two-dimensional interaction map showing contacts between ofloxacin and surrounding amino acid residues, with interaction types distinguished by color.

**Figure 12:**
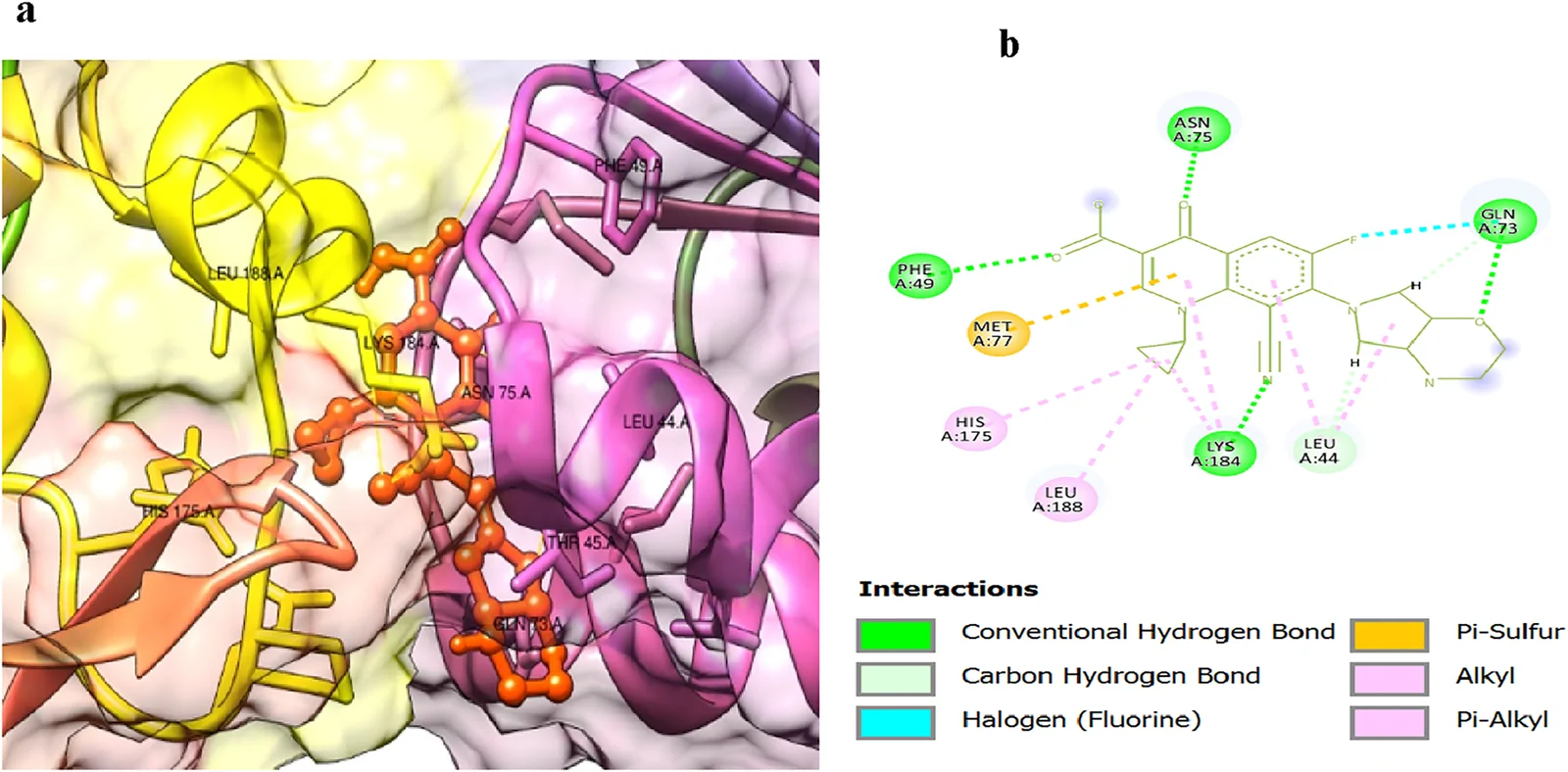
Predicted binding interactions between ciprofloxacin and the two-component system response regulator OmpR. (A) Three-dimensional view of ciprofloxacin within the protein binding pocket. (B) Two-dimensional interaction map showing contacts between ciprofloxacin and surrounding amino acid residues, with interaction types distinguished by color.

### MD analysis for protein-drug complex

Molecular Dynamics simulation was performed for a protein (Two-component system response regulator) docked with an antibiotic compound, Ciprofloxacin. MD analysis was performed for 100 ns. The results are shown in Figure 13. Figure 13(A): Red line often used to represent the RMSD of the protein backbone. This would track the structural deviations of the protein over time. The black line in the graph represents the RMSD of all protein atoms, including side chains. This tracks the extent to which the position of the whole protein changes relative to the initial structure. The red graph represents a completely stable structure with minimum deviation. Similarly, the black one has an initial phase (0–10 ns), and the RMSD quickly rises to about 1 nm, indicating significant conformational changes as the protein adjusts from its initial structure. Following an intermediate phase (10–60 ns), the RMSD values fluctuate around 1–1.5 nm. This suggests that the protein reached a relatively stable conformation with minor conformational changes. A later phase of (60–100 ns), where the RMSD shows more fluctuation and some larger deviations, particularly around 70 ns and 95 ns, indicates possible conformational changes or transitions to different stable states.

**Figure 13:**
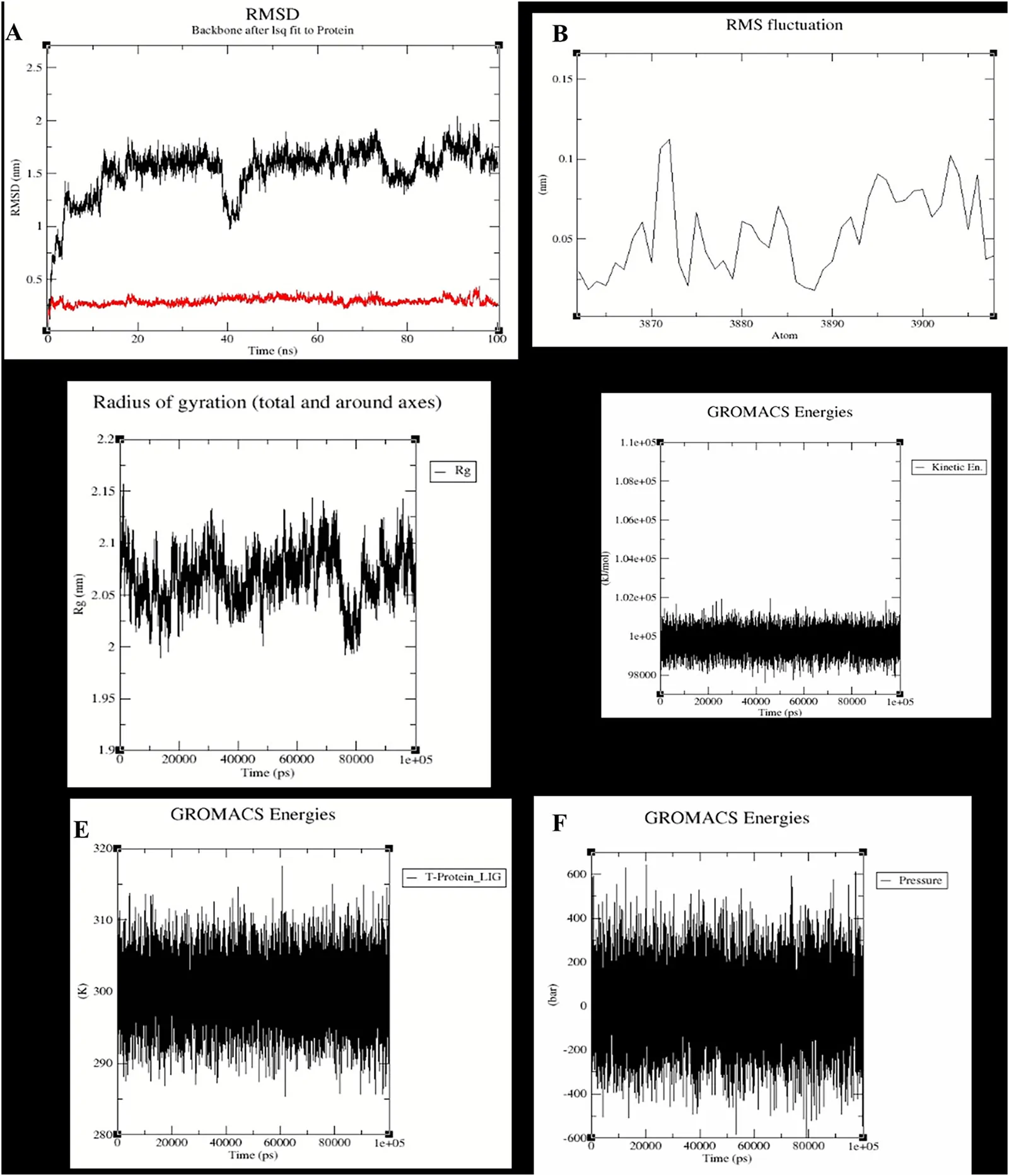
Overall, the results of the MD simulation analysis indicate a stable interaction of two-component system response regulator with ciprofloxacin, (A) the red line represents the stable RMSD of the protein backbone, while the black line reflects overall protein flexibility, including side chain movements., the red seems to be more stable, whereas some deviations can be observed in the black line, particularly around 70 ns and 95 ns. Plot (B) shows the RMSF value with a maximum value of 0.11 nm and a mean value of 0.05 nm. Furthermore, plot (C) predicted a compact structure with a maximum value for the Rg at 2.156 nm. The plots (D, E, F) for kinetic energy, temperature, and pressure further indicate the stability of the system.

Figure 13(B) presents the RMS fluctuation plot, illustrating the movement of each atom within the protein or molecule relative to its average position over time during the MD simulation. Peaks suggest regions with greater flexibility (e.g., loops or terminal ends of the protein), whereas flat regions indicate more stable parts of the structure (e.g., alpha helices or beta sheets). Overall, the graph represents some highly fluctuating atoms between the range of 3,870–3,875 with a maximum value of 0.11 nm and a mean value of 0.05 nm. Figure 13(C) shows the values of the Radius of Gyration (Rg), which is a measure of the compactness of a protein structure. It represents the distribution of the protein mass around its centre of mass. A structure with a smaller Rg is more compact, whereas a structure with a higher Rg is more spread. Rg had a maximum value of 2.156 and a mean value of 2.067. Figure 13(D) presents a graph of the kinetic energy with a mean value of 99,741.1 (KJ/mol) and a maximum value of 101,952 kJ/mol. Figure 13(E) and (F) show the temperature and pressure values of the system, respectively. The maximum temperature obtained was 317.5 K, while the mean temperature was 300.1 K with a standard deviation of 4.1. The pressure at 100ns is 643.0 bar.

The transcription terminator and anti-terminator proteins docked with ofloxacin were analyzed through a molecular dynamic simulation run for 100 ns. In Figure 14(A), the red line indicates RMSD for the backbone of the protein, and the black line indicates the RMSD of the protein side chains. In the first 20 ns, the RMSD values for both curves were relatively stable, with the black curve fluctuating around 1–2 nm, while the red curve showed minimal deviation (less than 1 nm). This suggests that the protein and specific segments remained stable during this initial phase. From 20 to 80 ns, the black curve shows more fluctuation between 1 and 3 nm, suggesting that the protein undergoes conformational changes. The red curve continued to exhibit minor fluctuations, indicating that the specific segment remained relatively stable compared to the rest of the protein. From 80 to 100 ns, the black curve indicates a significant increase in RMSD, reaching values of up to 6 nm near the end of the simulation, suggesting major conformational changes or instability in the protein structure. The red curve also shows some increase, but remains much lower, indicating that the specific segment is less affected by these changes. Furthermore, the spike in the black curve at approximately 60 ns may indicate a transient conformational change or some significant structural rearrangement in the protein.

**Figure 14:**
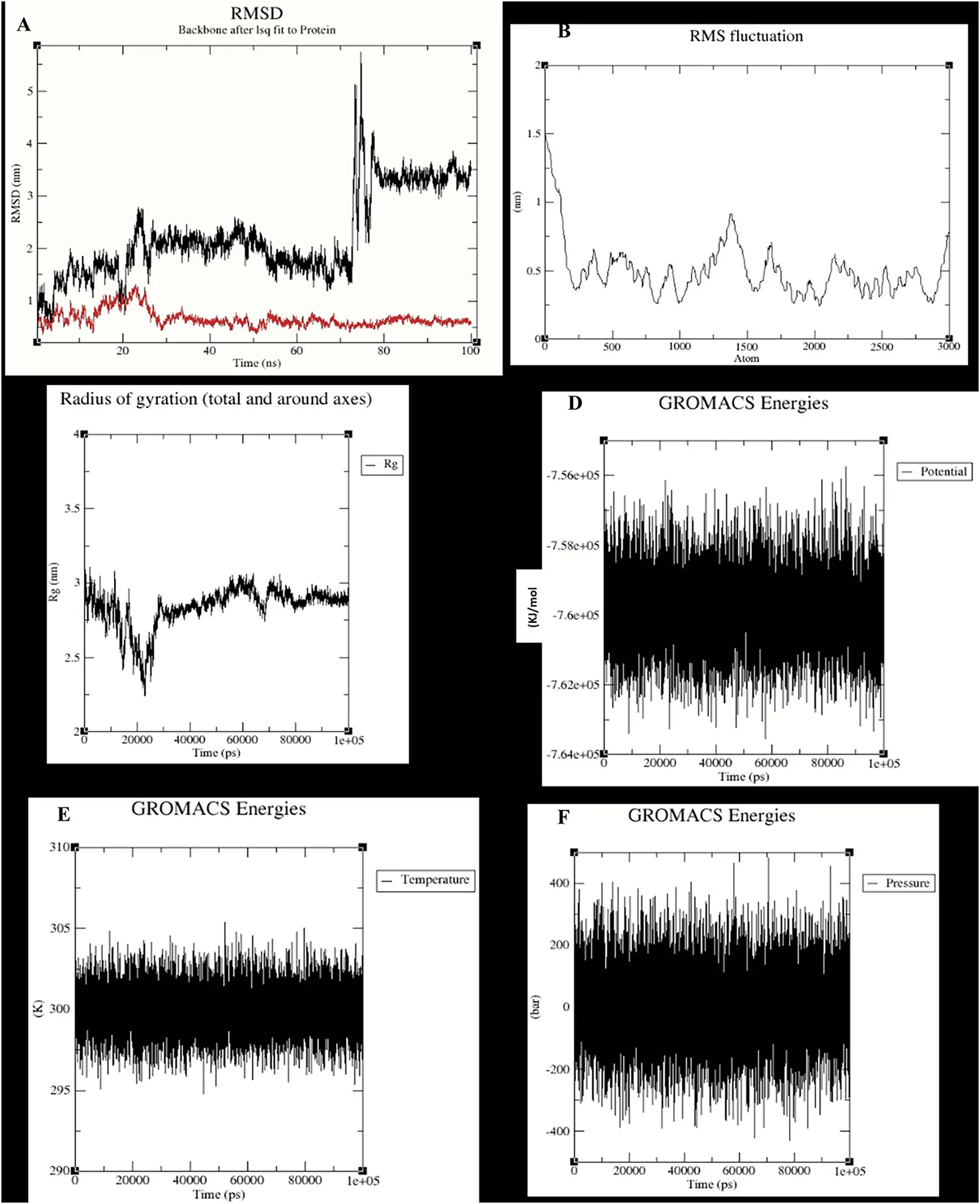
Represents results for MD simulation analysis, overall, the results indicate a stable interaction of transcription terminator and anti-terminator proteins with ofloxacin, (A) the red line represents the stable RMSD of the protein backbone, while the black line reflects overall protein flexibility, including side chain movements, the red seems to be more stable, whereas some deviations can be observed in black line particularly around 60 ns and 80–100 ns. Plot (B) presents the RMSF value with a maximum value of 1.2 nm and a mean value of 0.40 nm. Furthermore, plot (C) predicts a compact structure with a maximum value for radius of gyration at 2.42 nm. The plots (D, E, F), for potential energy, temperature and pressure, further indicate the stability of the system respectively.

Root Mean Square Fluctuation (RMSF) 14 (B). The peaks in the RMSF plot indicate highly flexible atoms. For example, the peaks around atom indices 500, 1,000, 2,500, and 3,000 suggest that these regions are more mobile than other parts of the protein. These peaks may correspond to loop regions, termini, or regions with low secondary structural stability. Similarly, the Troughs in the plot, where RMSF values are low (below 0.5 nm), represent more stable regions of the protein. These regions could correspond to alpha helices or beta sheets that are structurally constrained. The overall RMSF values mostly fluctuate between 0.3 and 0.6 nm, with only a few regions exceeding 1.0 nm. This suggests that the majority of the proteins remain relatively stable, with some localized flexibility. Moreover, the RMSF values were high at the beginning (atom indices close to 0) and at the end (near index 3,000), indicating that the N- and C-termini of the protein are more flexible compared to the core regions. This is a common observation in proteins, as termini are typically less constrained. The Rg plot 14(C) represents fluctuation around 2.3 nm during the initial phase (up to approximately 40,000 ps), suggesting that the protein maintains a relatively consistent compact structure. From 40,000–80,000 ps the Rg values exhibited increased fluctuations, reaching a maximum close to 2.4 nm. This indicates that the protein expands or temporarily adopts different conformations. Furthermore, after 80,000 ps the Rg values decrease below 2.2 nm and fluctuate at lower values towards the end, suggesting that the protein structure becomes more compact or undergoes a folding event. Plot 14(D), illustrated here, correlates with the GROMACS energy graph depicting the total potential energy of a molecular system as a time-dependent function of a molecular dynamics simulation, which lasts up to 100 ns. The y-axis represents the potential energy in kilojoules per mole (kJ/mole) and ranges from approximately −5.84 × 10^9^ and −5.9 × 10^9^ kJ/mol. These fluctuations denote normal atomic interactions, whereas a relatively constant energy range suggests that the system has achieved equilibrium. The maintenance of the energy band shows that the simulation reaches a stable state, enabling more advanced structural or thermodynamic analyses. Figure 14(E) and (F) show the temperature and pressure values of the system, respectively. The maximum temperature obtained was 306K. The pressure at 100ns was 549.173 bar.

The translation initiation factor docked with ciprofloxacin was run for MD analysis for 100 ns. The Figure 15(A) RMSD plot the red line indicate RMSD for the backbone of the protein and the black line indicate RMSD of protein side chains, the black line shows an initial rise in RMSD, leveling off around 2 nm by 40 ns, which suggests that the system undergoes some conformational changes but then stabilizes. At around 80 ns, there’s a sharp increase in RMSD to ∼5 nm, indicating a significant conformational change or fluctuation, after which it stabilizes at around 4 nm. This suggests a structural event, such as unfolding or transition to a different conformational state. Furthermore, 15(B) RMSF (Root Mean Square Fluctuation), atoms near the start (0–100) atoms and end (around 3,000) of the sequence show higher RMSF values, reaching close to 1.5–2.0 nm. This suggests that the terminal regions of the proteins are flexible. Moreover, between atom indices 500 and 2,500, the RMSF values are lower (around 0.5 nm), indicating that these atoms are part of a more stable or rigid region of the protein. However, there are a few peaks around 1,500 and 2000, which correspond to areas of increased flexibility, possibly loop regions or regions not involved in maintaining the core structure.

**Figure 15:**
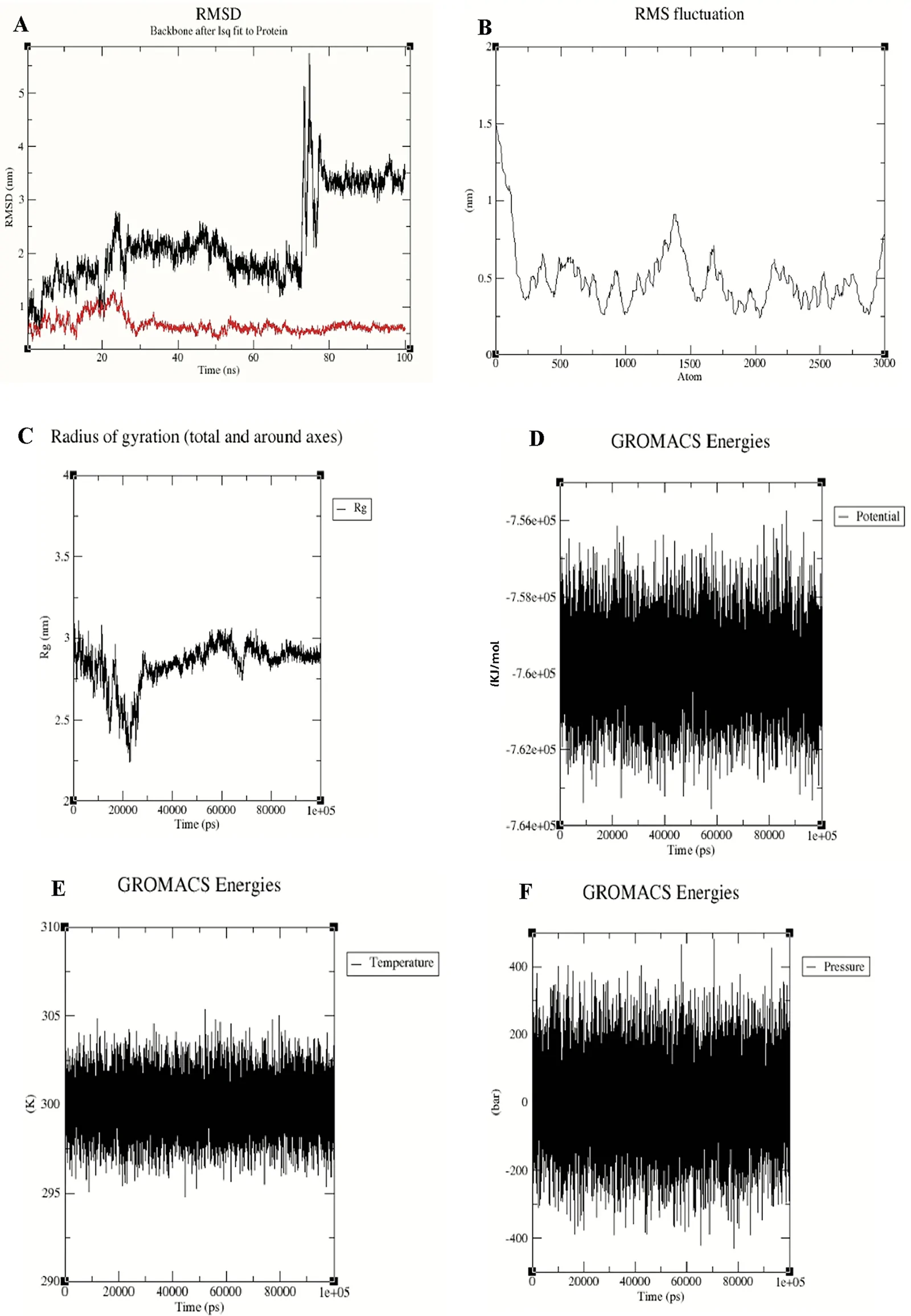
Represents results for MD simulation analysis of translation initiation factor with ciprofloxacin. Overall, the results indicate a stable interaction of protein with its ligand. (A) The red line represents the RMSD of the protein backbone, while the black line reflects overall protein flexibility, including side chain movements. Whereas some deviations can be observed in the black line, particularly around 80 ns, a sharp conformational change is observed. Plot (B) presents the RMSF value with a maximum value of 1.5 nm and a mean value of 0.40 nm. Furthermore, plot (C) predicts a compact structure with a maximum value for radius of gyration at 3.1 nm. The plots (D, E, F), for potential energy, temperature and pressure, further indicate the stability of the system, respectively.

For Rg 15(C), initially 0–30,000 ps, the Rg fluctuates around 3 nm, showing that the system might be exploring different conformations but remains fairly compact. There is a slight drop in the Rg value at 30,000–50,000 ps, dipping closer to 2.5 nm, suggesting the system becomes more compact during this period. In a later phase, 50,000–100,000 ps. The Rg fluctuates between 2.7 and 3.0 nm, indicating that after some initial conformational rearrangements, the system stabilizes. There are minor fluctuations, which are expected in a dynamic system, but the overall trend suggests stability in the molecule’s compactness after 50 ns. Furthermore, Figure 15(D) represents potential energy variation between −7.64 × 10^5^ and −7.56 × 10^5^ units. Minor energy changes indicate that the system has reached equilibrium and operates as anticipated in the simulation. Similarly, Figure 15(E) and (F) depict the temperature and pressure values of the system. The maximum temperature obtained was 305.5 K. The pressure at 100 ns was 600.0 bar.

## Discussion

*Yersinia enterocolitica* continues to be a clinically significant gastrointestinal pathogen that has demonstrated increased multidrug resistance in recent years [1], 41]. This growing resistance emphasizes the need for novel treatment discovery methodologies [42], 43], particularly genome-driven methods capable of identifying conserved and important bacterial targets [44]]. Previous research on *Y. enterocolitica* has been focused on epidemiology, virulence factors, and phenotypic antibiotic resistance. In contrast, we present an integrated, genome-wide in-silico workflow that goes beyond descriptive investigations. This study provides a thorough method for prioritizing therapeutically relevant proteins by analyzing 22 complete genomes, comparing core sets derived from Roary and EDGAR, using subtractive genomics and essentiality filtering, and validating target–drug interactions through homology modeling, druggability scoring, docking, and molecular dynamics simulation. To our knowledge, such a multi-layered computational paradigm has never been used to *Y. enterocolitica*, and it provides mechanistic insights on conserved vulnerabilities that are still targetable despite rising drug resistance.

In this study, we used subtractive genomics techniques to identify possible drug candidates that could serve as a leading treatment for the future. Pangenome analysis was performed to identify the core genomes that are potential therapeutic candidates [45]. The number of core genes resulting from Roary for all 22 complete strains was 2,793, whereas EDGAR identified 3,025 core genes because of algorithmic differences using a heuristic approach; however, they might not be as accurate as EDGAR. CD-HIT was used to perform the first clustering step, while a graph-based method was used to further refine the clusters. In contrast, the EDGAR orthologous clustering web service employs an alignment-based approach by utilizing either BLAST or DIAMOND, which is more precise and sensitive for comparing and matching sequences. This often leads to a more accurate core gene set in most cases when new genes are added, resulting in a more precise, larger core gene set. The other possible cause might be variations in the software being used, as well as the updates that are applied to the program. Common updates may contain better algorithms or corrections for some bugs that change the fundamental process of determining genes. It is clear that the EDGAR algorithm significantly speeds up the learning rate in data mining without compromising accuracy compared with other distributed algorithms [46].

The number of non-host homologous proteins identified was 2,267. Non-host homologous proteins are important during drug and vaccine candidate identification because they provide added value in therapeutic development as they have minimal chances of cross-reactivity and side effects when targeted by drugs and vaccines [47]. In addition, 29 essential proteins have been identified [48], which are crucial for pathogen survival and are prime targets for therapeutic development owing to their involvement in critical cellular functions and reduced likelihood of developing resistance. Furthermore, those essential genes were categorized based on their topology into two groups i.e., membrane and cytoplasmic, the result indicated 1 membrane protein and 9 cytoplasmic proteins.

Moreover, cytoplasmic proteins were analyzed for their physicochemical properties and were selected as potential drug targets because they play essential roles in bacterial growth, survival, and virulence, making them promising candidates for therapeutic intervention [49]. The choice of cytoplasmic proteins is based on various factors, such as molecular weight, molecular function, cellular localization, pathway analysis, and virulence, with proteins with a molecular weight <100 kDa considered optimal therapeutic targets [48]. Additionally, the localization of proteins in the cytoplasm makes them accessible targets for drug development because they are more amenable to interactions with drug molecules than extracellular or membrane-bound proteins [50]. Nine cytoplasmic proteins were subsequently modeled using SWISS-MODEL. This online server was specifically designed for automated comparative modeling of three-dimensional (3D) protein structures. Through this web tool, researchers can readily obtain 3D structures of their target proteins [51]. The DoGSite Scorer was used to evaluate the druggability of the acquired 3D structures. This tool can identify potential binding pockets and subpockets within a protein of interest. It predicts catalytic pockets and assigns a druggability score on a scale of 0–1. A score closer to 1 indicates a highly druggable protein pocket, which is considered the most favorable for targeting potential therapeutics [52]. Moreover, the protein structure was validated using Galaxy Refine, Ramachandran plot, and ERRAT score to ensure its accuracy and reliability. Galaxy Refine, a web-based refinement tool, generates five possible models of the target protein.

These models were subsequently analyzed to identify the most optimal one based on the evaluation criteria, including the Ramachandran plot and ERRAT score. Ramachandran plot analysis confirmed the excellent quality of the model, with a quality score exceeding 95 %. These proteins were docked with a group of fluoroquinolone compounds, which show promising antibacterial activity against gram-negative bacteria and potential for treating systemic infections when administered orally in well-tolerated doses [53]. Furthermore, fluoroquinolones can alter protein conformation and DNA cleavage, leading to different physiological conditions, depending on the location of the enzyme [54]. Fluoroquinolones are synthetic antibacterial agents that possess their activity by inhibiting the functions of DNA gyrase and topoisomerase IV, which are crucial for bacterial DNA replication and repair processes. The broad-spectrum action of these antibiotics against various bacteria is well recognized. According to Ropón-Palacios, Chenet-Zuta [55], interactions are considered acceptable when the Root Mean Square Deviation (RMSD) score is equal to or less than 2.0. The 30 ligands selected from the literature indicated that our bacteria were already resistant to them, and we aimed to repurpose these compounds using *in silico* techniques. Each protein candidate was explored for its efficacy and has been mentioned in detail, along with its best-docked target.

Research findings indicate that ciprofloxacin, in addition to other antibiotics, such as trimethoprim-sulfamethoxazole, aminoglycosides, third-generation cephalosporins, and tetracyclines, has demonstrated efficacy against *Y. enterocolitica* infections. Conversely, organisms commonly exhibit resistance to first-generation cephalosporins and a wide range of penicillins [56]. *Y. enterocolitica* has occasionally been reported to be resistant to macrolides and fluoroquinolones [57]. After docking, the results were analyzed for each candidate, and the Two-component system response regulator, OmpR, showed the best docking with ciprofloxacin. The function of the Two-component system response regulator OmpR is to regulate various cellular processes in bacteria [58]. OmpR is a key component of the two-component regulatory system and plays a crucial role in bacterial signal transduction and adaptation to environmental changes. OmpR acts as a response regulator, influencing gene expression in response to external stimuli such as osmolarity, pH, and temperature. Specifically, OmpR regulates virulence factors, biofilm formation, flagellar expression, and mucoviscosity in various bacterial species such as *Escherichia coli*, *Shigella flexneri*, and *Y. enterocolitica* [59], 60]. Furthermore, our analysis highlighted its efficient response to ciprofloxacin, indicating that it is a potential drug candidate with a docking score of −7.4 kcal/mol.

Furthermore, the Large ribosomal subunit protein uL13, also known as RPL13, has been identified as a critical regulator of IRES-driven translation during viral infections, suggesting its potential as a target for antiviral drug development. It plays a crucial role in the internal ribosome entry site (IRES)-driven translation of viruses such as foot-and-mouth disease virus (FMDV) [61]. Our docking results indicated that the large ribosomal subunit protein uL13 is a potential drug candidate for nalidixic acid, with a docking score of −7.8 kcal/mol. Nalidixic acid inhibits the activity of DNA gyrase, a type II topoisomerase that plays a crucial role in DNA replication and transcription [39], 62]. The observed increase in resistance to nalidixic acid is considerable and requires careful consideration when handling *Y. enterocolitica* infections [40]. Similarly, the role of phosphoheptose isomerase, also known as GmhA [63], indicated its crucial role in lipopolysaccharide biosynthesis in bacteria such as *Leptospira*. Moreover, the presence of phosphoheptose isomerase has been noted in metabolic pathways of bacteria, such as *Ralstonia eutropha* H16, which is involved in sugar metabolism [64]. From its diverse function and our study, this protein proved to be an effective drug candidate and showed a potential response to norfloxacin with a docking score of −5.4 kcal/mol [65]. Furthermore, Andualem and Geyid [66] studied the susceptibility of *Y. enterocolitica* to Norfloxacin, but the specific target proteins were not mentioned.

In addition, Protein-export protein SecB, Shikimate kinase 1, and Translation initiation factor IF-3 showed good docking scores of −7.3 kcal/mol, −4.5 kcal/mol, and −5.4 kcal/mol with Ciprofloxacin, respectively. Protein-export protein SecB has been extensively studied for its role as a chaperone involved in protein export processes in bacteria such as *E. coli* [67]. Similarly, the *Y. enterocolitica* Sec system, which includes SecB, is a potential antibiotic drug target due to its involvement in protein export processes [68]. Because ciprofloxacin has previously been reported to be resistant to several gram-negative bacteria, it can now serve as a sensitive and potential antibiotic in our proposed study. Shikimate kinase also showed good docking scores of −4.9 kcal/mol and interactions with Balofloxacin, which is an antibiotic that exerts its mechanism of action by targeting a bacterial enzyme called DNA gyrase. This action inhibits the DNA gyrase and topoisomerase IV enzymes, which are essential for maintaining the integrity of bacterial DNA. By interfering with these enzymes, Balofloxacin prevents bacterial cells from dividing and repairing, ultimately leading to their death National Center for Biotechnology Information, 2024). PubChem Compound Summary for CID 65958, Balofloxacin. Retrieved May 20, 2024, from (https://pubchem.ncbi.nlm.nih.gov/compound/Balofloxacin). Similarly, Ofloxacin played the same role as balofloxacin and showed a good docking score of −5.1 kcal/mol.

Moreover, the Translation initiation factor IF-3 has also been identified as an efficient drug candidate for ciprofloxacin. In addition, Gatifloxacin, Rufloxacin, Besifloxacin, and Grepafloxacin were studied for their potential antibiotic activity against *Y. enterocolitica* [69]. Gatifloxacin exhibits enhanced activity against *Y. enterocolitica*, with low MIC (90) values against various pathogens, including *Yersinia* species. In contrast, besifloxacin, a fourth-generation ophthalmic fluoroquinolone, demonstrates broad-spectrum antibiotic activity against ocular bacterial pathogens, including *Yersinia* species [70].

Glutaredoxin (Grx) has been identified as a potential drug target owing to its various roles in physiological and pathological conditions [71]. It is a small protein containing an active-site cysteine pair that catalyzes glutathione-dependent redox regulation via glutathionylation and deglutathionylation. Glutaredoxin showed the best docking results, with grepafloxacin having a docking score of-7.2 kcal/mol. Grepafloxacin exerts its antibacterial activity by inhibiting bacterial topoisomerase II (DNA gyrase) and topoisomerase IV, which are essential enzymes for the duplication, transcription, and repair of bacterial DNA. By inhibiting these enzymes, grepafloxacin disrupts bacterial DNA replication, leading to bacterial cell death [72]. Moreover, Grepafloxacin has been extensively studied to assess its efficacy and safety compared to other antibiotics. Research indicates that when used at a dose of 400 or 600 mg once daily, grepafloxacin is as effective as ciprofloxacin (500 mg twice daily) for treating acute bacterial exacerbations of chronic bronchitis [73]. These data demonstrate the effectiveness of grepafloxacin as a potential drug compound for resistant *Y. enterocolitica*.

The small ribosomal subunit protein uS5, known for its essential role in ribosome assembly and translational fidelity [74], also plays a crucial role in extra-ribosomal functions, making it a target for drug development in *Y. enterocolitica* [75]. Studies have identified a druggable pocket within uS5, particularly in the C-terminal helical bundles and helices 9 and 5, which are essential for its function as a translocator in bacterial infections [76]. By targeting this pocket, small-molecule inhibitors such as Salicylidene Acyl Hydrazide derivatives and phenoxyacetamide derivatives have shown promising interactions with uS5, disrupting its oligomerization and interactions with other proteins such as YspB, thereby inhibiting the formation of functional translocons crucial for *Y. enterocolitica* infection [77]. This highlights the potential of uS5 as a drug candidate for combating *Y. enterocolitica* infections by interfering with its essential translocation mechanisms. Moreover, the highest docking score was observed for fleroxacin. The activity of fleroxacin is comparable to that of other quinolones, such as ciprofloxacin, norfloxacin, and ofloxacin, albeit slightly less potent than ciprofloxacin. Additionally, fleroxacin has shown high clinical and bacteriological efficacy rates, with minimal adverse effects observed during treatment, making it a promising drug candidate for combating bacterial infections, such as *Y. enterocolitica*. Therefore, the drug’s ability to target small ribosomal subunit protein uS5 contributes to its mechanism of action, disrupting bacterial protein synthesis and ultimately leading to bacterial cell death.

The transcription terminator and anti-terminator protein NusG play crucial roles in gene expression regulation by facilitating transcription termination and anti-termination processes. In *Y. enterocolitica*, NusG acts as a potential drug candidate because of its ability to modulate RNA polymerase activity, influencing the termination of transcription. Studies have shown that NusG interacts with RNA polymerase to regulate pausing and termination, preventing the formation of terminator RNA hairpins and promoting transcription elongation [78]. In the current study, we found a good interaction of ofloxacin with the transcription terminator and antiterminator protein NusG, with a docking score of −6.3 kcal/mol, consequently, indicating its potential as a drug target. Moreover, Ofloxacin, an antimicrobial drug, is used to treat various bacterial infections. It inhibits the supercoiling activity of bacterial DNA gyrase, halts DNA replication, and acts on DNA gyrase and topoisomerase IV, preventing the excessive supercoiling of DNA during replication or transcription.

Additionally, the Molecular Dynamic Simulation conducted by GROMACS revealed a stable configuration for the prioritized proteins and their ligands docked complex, and we selected transcription termination/antitermination protein, two-component system response regulator, and translation initiation factor for the MD analysis because they play fundamental roles in the major cellular functions that include such enormous tasks like the regulation of gene expression, signaling mechanism, and protein synthesis, which are critical for bacterial survival and virulence; hence, they represent high-value drug targets. Moreover, their structural features, such as potential binding sites and conformational flexibility, are ideal for high-level molecular dynamics simulations.

## Conclusions

This study highlights the critical need for new therapeutic strategies against *Y. enterocolitica* given its increasing antibiotic resistance. By employing subtractive genomics and drug repurposing studies, essential non-host homologous proteins were identified, offering promising drug targets. Pangenome analysis revealed 3,025 core genes, and subsequent protein modeling identified nine key cytoplasmic proteins. Docking studies with fluoroquinolones identified strong interactions, particularly for the Two-component system response regulator OmpR with ciprofloxacin and the Large ribosomal subunit protein uL13 with nalidixic acid. These findings provide a foundation for developing effective treatments against *Y. enterocolitica*, thereby addressing the urgent need for alternative therapeutic options to combat this pathogen.

## References

[1] Yue Y, Zheng J, Sheng M, Liu X, Hao Q, Zhang S (2023). Public health implications of Yersinia enterocolitica investigation: an ecological modeling and molecular epidemiology study. Infect Dis Poverty.

[2] Hordofa DL, Nuguse DA (2022). Review on yersiniosis and its public health importance. Int J Vet Sci Anim Husbandry.

[3] Bottone EJ (1999). Yersinia enterocolitica: overview and epidemiologic correlates. Microb Infect.

[4] Mollaret H (1987). A personal view of the history of the genus Yersinia. Contrib Microbiol Immunol.

[5] Morka K, Wałecka-Zacharska E, Schubert J, Dudek B, Woźniak-Biel A, Kuczkowski M (2021). Genetic diversity and distribution of virulence-associated genes in Y. enterocolitica and Y. Enterocolitica-like isolates from humans and animals in Poland. Pathogens.

[6] Schmühl C, Beckstette M, Heroven AK, Bunk B, Spröer C, McNally A (2019). Comparative transcriptomic profiling of Yersinia enterocolitica O: 3 and O: 8 reveals major expression differences of fitness-and virulence-relevant genes indicating ecological separation. mSystems.

[7] Aleksić S (1995). Occurrence of Y. enterocolitica antigens O: 3, O: 9 and O: 8 in different Yersinia species, their corresponding H antigens and origin. Contrib Microbiol Immunol.

[8] Matsumoto H, Young GM (2009). Translocated effectors of Yersinia. Curr Opin Microbiol.

[9] McNally A, Cheasty T, Fearnley C, Dalziel R, Paiba G, Manning G (2004). Comparison of the biotypes of Yersinia enterocolitica isolated from pigs, cattle and sheep at slaughter and from humans with yersiniosis in Great Britain during 1999–2000. Lett Appl Microbiol.

[10] Wang X, Cui Z, Jin D, Tang L, Xia S, Wang H (2009). Distribution of pathogenic Yersinia enterocolitica in China. Eur J Clin Microbiol Infect Dis.

[11] Bottone EJ (2015). Clinical microbiology: open access. ..

[12] Seakamela EM, Diseko L, Malatji D, Makhado L, Motau M, Jambwa K (2022). Characterisation and antibiotic resistance of Yersinia enterocolitica from various meat categories, South Africa. Onderstepoort J Vet Res.

[13] Didkowska A, Orłowska B, Krajewska-Wędzina M, Krzysiak M, Bruczyńska M, Wiśniewski J (2022). Intra-palpebral tuberculin skin test and interferon gamma release assay in diagnosing tuberculosis due to Mycobacterium caprae in European Bison (Bison bonasus). Pathogens.

[14] Jamali H, Paydar M, Radmehr B, Ismail S (2015). Prevalence, characterization, and antimicrobial resistance of Yersinia species and Yersinia enterocolitica isolated from raw milk in farm bulk tanks. J Dairy Sci.

[15] Seemann T (2014). Prokka: rapid prokaryotic genome annotation. Bioinformatics.

[16] Page AJ, Cummins CA, Hunt M, Wong VK, Reuter S, Holden MT (2015). Roary: rapid large-scale prokaryote pan genome analysis. Bioinformatics.

[17] Blom J, Albaum SP, Doppmeier D, Pühler A, Vorhölter F-J, Zakrzewski M (2009). EDGAR: a software framework for the comparative analysis of prokaryotic genomes. BMC Bioinf.

[18] Rizwan M, Naz A, Ahmad J, Naz K, Obaid A, Parveen T (2017). VacSol: a high throughput in silico pipeline to predict potential therapeutic targets in prokaryotic pathogens using subtractive reverse vaccinology. BMC Bioinf.

[19] Zhang R, Ou HY, Zhang CT (2004). DEG: a database of essential genes. Nucleic Acids Res.

[20] Clarke S, Dillon J (2011). The cell culture laboratory. Animal Cell Culture: Essential Methods.

[21] Desai S, Tahilramani P, Patel D, Meshram D, Patel P (2017). In silico prediction and docking of tertiary structure of protein X, multifunctional protein of hepatitis B virus (Hbv). Indo Am J Pharm Res.

[22] Qureshi H, Basheer A, Sajjad W, Faheem M, Babar Jamal S (2024). An integrated in-silico approach for drug target identification in human pathogen Shigella dysenteriae. PLoS One.

[23] Di Muzio E, Toti D, Polticelli F (2017). DockingApp: a user friendly interface for facilitated docking simulations with AutoDock Vina. J Comput Aided Mol Des.

[24] Trott O, Olson AJ (2010). AutoDock Vina: improving the speed and accuracy of docking with a new scoring function, efficient optimization, and multithreading. J Comput Chem.

[25] da Silva TU, Pougy KC, Albuquerque MG, da Silva Lima CH, Machado SP (2022). Development of parameters compatible with the CHARMM36 force field for [Fe4S4] 2+ clusters and molecular dynamics simulations of adenosine-5′-phosphosulfate reductase in GROMACS 2019. J Biomol Struct Dyn.

[26] Izadi S, Anandakrishnan R, Onufriev AV (2014). Building water models: a different approach. J Phys Chem Lett.

[27] Vanommeslaeghe K, Hatcher E, Acharya C, Kundu S, Zhong S, Shim J (2010). CHARMM general force field: a force field for drug-like molecules compatible with the CHARMM all-atom additive biological force fields. J Comput Chem.

[28] Ross GA, Rustenburg AS, Grinaway PB, Fass J, Chodera JD (2018). Biomolecular simulations under realistic macroscopic salt conditions. J Phys Chem B.

[29] Helal MA, Shouman S, Abdelwaly A, Elmehrath AO, Essawy M, Sayed SM (2022). Molecular basis of the potential interaction of SARS-CoV-2 spike protein to CD147 in COVID-19 associated-lymphopenia. J Biomol Struct Dyn.

[30] Golo V, Shaĭtan K (2002). Dynamic attractor for the Berendsen thermostat an the slow dynamics of biomacromolecules. Biofizika.

[31] Tuble SC, Anwar J, Gale JD (2004). An approach to developing a force field for molecular simulation of martensitic phase transitions between phases with subtle differences in energy and structure. J Am Chem Soc.

[32] Darden T, York D, Pedersen L (1993). Particle mesh Ewald: an N· log (N) method for Ewald sums in large systems. J Chem Phys.

[33] Hess B, Bekker H, Berendsen HJ, Fraaije JG (1997). LINCS: a linear constraint solver for molecular simulations. J Comput Chem.

[34] Páll S, Hess B (2013). A flexible algorithm for calculating pair interactions on SIMD architectures. Comput Phys Commun.

[35] Best RB, Zhu X, Shim J, Lopes PE, Mittal J, Feig M (2012). Optimization of the additive CHARMM all-atom protein force field targeting improved sampling of the backbone ϕ, ψ and side-chain χ1 and χ2 dihedral angles. J Chem Theor Comput.

[36] Humphrey W, Dalke A, Schulten K (1996). VMD: visual molecular dynamics. J Mol Graph.

[37] DeLano WL (2002). Pymol: an open-source molecular graphics tool. CCP4 Newsl Protein Crystallogr.

[38] Capilla S, Goni P, Rubio M, Castillo J, Millan L, Cerda P (2003). Epidemiological study of resistance to nalidixic acid and other antibiotics in clinical Yersinia enterocolitica O: 3 isolates. J Clin Microbiol.

[39] Sánchez-Céspedes J, Navia MM, Martínez R, Orden B, Millán R, Ruiz J (2003). Clonal dissemination of Yersinia enterocolitica strains with various susceptibilities to nalidixic acid. J Clin Microbiol.

[40] Hulankova R (2022). Higher resistance of Yersinia enterocolitica in comparison to Yersinia pseudotuberculosis to antibiotics and Cinnamon, oregano and thyme essential oils. Pathogens.

[41] Basharat Z, Alghamdi YS, Mashraqi MM, Ogaly HA, Al-Zahrani FA, Wei CR (2025). Proteome mining of Yersinia Enterocolitica for drug targets and computational inhibitor identification with ADMET, anti-inflammation potential and formulation characteristics. BioData Min.

[42] Black RE, Jackson RJ, Tsai T, Medvesky M, Shayegani M, Feeley JC (1978). Epidemic Yersinia enterocolitica infection due to contaminated chocolate milk. N Engl J Med.

[43] Hering NA, Richter JF, Krug SM, Günzel D, Fromm A, Bohn E (2011). Yersinia enterocolitica induces epithelial barrier dysfunction through regional tight junction changes in colonic HT-29/B6 cell monolayers. Lab Invest.

[44] Al-Rawe A, Al-Jomaily O, Yousif Y, Shaban S, Suleiman A (2023). Comparative genomics, phylogenetic and functional analysis of Yersinia enterocolitica, a gastrointestinal pathogen, with other soil-borne bacteria causing diseases. Mikrobiol Zh.

[45] Aslam M, Shehroz M, Shah M, Khan MA, Afridi SG, Khan A (2020). Potential druggable proteins and chimeric vaccine construct prioritization against Brucella melitensis from species core genome data. Genomics.

[46] Rodríguez M, Escalante DM, Peregrín A (2011). Efficient distributed genetic algorithm for rule extraction. Appl Soft Comput.

[47] Solanki V, Tiwari V (2018). Subtractive proteomics to identify novel drug targets and reverse vaccinology for the development of chimeric vaccine against Acinetobacter baumannii. Sci Rep.

[48] Naorem RS, Pangabam BD, Bora SS, Goswami G, Barooah M, Hazarika DJ (2022). Identification of putative vaccine and drug targets against the methicillin-resistant Staphylococcus aureus by reverse vaccinology and subtractive genomics approaches. Molecules.

[49] Qureshi NA, Bakhtiar SM, Faheem M, Shah M, Bari A, Mahmood HM (2021). Genome-based drug target identification in human pathogen Streptococcus gallolyticus. Front Genet.

[50] Ahamed NA, Panneerselvam A, Arif IA, Abuthakir MHS, Jeyam M, Ambikapathy V (2021). Identification of potential drug targets in human pathogen Bacillus cereus and insight for finding inhibitor through subtractive proteome and molecular docking studies. J Infect Public Health.

[51] Nielsen M, Lundegaard C, Lund O, Petersen TN (2010). CPHmodels-3.0 – remote homology modeling using structure-guided sequence profiles. Nucleic Acids Res.

[52] Tiwari S, Jamal SB, Hassan SS, Carvalho PV, Almeida S, Barh D (2017). Two-component signal transduction systems of pathogenic bacteria as targets for antimicrobial therapy: an overview. Front Microbiol.

[53] Mandal D, Mandal D, Basu A (2020). YspD: a potential therapeutic target for drug design to combat Yersinia enterocolitica infection. Int J Pept Res Therapeut.

[54] Drlica K (1999). Mechanism of fluoroquinolone action. Curr Opin Microbiol.

[55] Ropón-Palacios G, Chenet-Zuta ME, Olivos-Ramirez GE, Otazu K, Acurio-Saavedra J, Camps I (2020). Potential novel inhibitors against emerging zoonotic pathogen Nipah virus: a virtual screening and molecular dynamics approach. J Biomol Struct Dyn.

[56] Kot B, Piechota M, Jakubiak K (2017). Virulence genes and antibiotic resistance of Yersinia enterocolitica strains isolated from children. Eur J Biomed Res.

[57] Koskinen J, Ortiz-Martínez P, Keto-Timonen R, Joutsen S, Fredriksson-Ahomaa M, Korkeala H (2022). Prudent antimicrobial use is essential to prevent the emergence of antimicrobial resistance in Yersinia enterocolitica 4/O: 3 strains in pigs. Front Microbiol.

[58] Ma W, Wang X, Zhang W, Hu X, Yang J-L, Liang X (2023). Two-component system response regulator ompR regulates mussel settlement through exopolysaccharides. Int J Mol Sci.

[59] Brzóstkowska M, Raczkowska A, Brzostek K (2012). OmpR, a response regulator of the two-component signal transduction pathway, influences inv gene expression in Yersinia enterocolitica O9. Front Cell Infect Microbiol.

[60] Shimada T, Takada H, Yamamoto K, Ishihama A (2015). Expanded roles of two‐component response regulator OmpR in Escherichia coli: genomic SELEX search for novel regulation targets. Genes Cells.

[61] Han S, Sun S, Li P, Liu Q, Zhang Z, Dong H (2020). Ribosomal protein L13 promotes IRES-driven translation of foot-and-mouth disease virus in a helicase DDX3-dependent manner. J Virol.

[62] Tavassoli M, Afshari A, Drăgănescu D, Arsene AL, Burykina TI, Rezaee R (2018). Antimicrobial resistance of Yersinia enterocolitica in different foods: a review. FARMACIA.

[63] Umamaheswari A, Pradhan D, Hemanthkumar M (2010). Identification of potential Leptospira phosphoheptose isomerase inhibitors through virtual high-throughput screening. Genom Proteom Bioinform.

[64] Saini V, Raghuvanshi S, Khurana JP, Ahmed N, Hasnain SE, Tyagi AK (2012). Massive gene acquisitions in Mycobacterium indicus pranii provide a perspective on mycobacterial evolution. Nucleic Acids Res.

[65] Shungu DL, Weinberg E, Gadebusch HH (1983). In vitro antibacterial activity of norfloxacin (MK-0366, AM-715) and other agents against gastrointestinal tract pathogens. Antimicrob Agents Chemother.

[66] Andualem B, Geyid A (2005). Antimicrobial responses of Yersinia enterocolitica isolates in comparison to other commonly encountered bacteria that causes diarrhoea. East Afr Med J.

[67] Lilly AA, Crane JM, Randall LL (2009). Export chaperone SecB uses one surface of interaction for diverse unfolded polypeptide ligands. Protein Sci.

[68] Hardy S, Randall L, Creighton T, Laskey RA, Lorimer GH (1993). Recognition of ligands by SecB, a molecular chaperone involved in bacterial protein export. Phil Trans Roy Soc Lond B Biol Sci.

[69] Blondeau JM (2000). Gatifloxacin: a new fluoroquinolone. Expert Opin Invest Drugs.

[70] Singh CL, Singh A, Kumar S, Majumdar D (2014). Besifloxacin the fourth generation fluoroquinolone: a review. J Drug Deliv Therapeut.

[71] Ogata FT, Branco V, Vale FF, Coppo L (2021). Glutaredoxin: discovery, redox defense and much more. Redox Biol.

[72] Marriott MS (1998). Grepafloxacin: microbiological properties. Clin Microbiol Infection.

[73] Chodosh S, Lakshminarayan S, Swarz H, Breisch S (1998). Efficacy and safety of a 10-day course of 400 or 600 milligrams of grepafloxacin once daily for treatment of acute bacterial exacerbations of chronic bronchitis: comparison with a 10-day course of 500 milligrams of ciprofloxacin twice daily. Antimicrob Agents Chemother.

[74] Landry-Voyer A-M, Mir Hassani Z, Avino M, Bachand F (2023). Ribosomal protein uS5 and friends: protein–protein interactions involved in ribosome assembly and beyond. Biomolecules.

[75] Kamath D, Gregory ST, O’Connor M (2017). The loop 2 region of ribosomal protein uS5 influences spectinomycin sensitivity, translational fidelity, and ribosome biogenesis. Antimicrob Agents Chemother.

[76] Steiner A, Favre S, Mack M, Hausharter A, Pillet B, Hafner J (2023). Dissecting the nuclear import of the ribosomal protein Rps2 (uS5). Biomolecules.

[77] Kirthi N, Roy-Chaudhuri B, Kelley T, Culver GM (2006). A novel single amino acid change in small subunit ribosomal protein S5 has profound effects on translational fidelity. RNA.

[78] Walshe JL, Siddiquee R, Patel K, Ataide SF (2022). Structural characterization of the ANTAR antiterminator domain bound to RNA. Nucleic Acids Res.

